# Modeling the weaning diet of piglets with fermented feed material: effects on growth performance and health parameters

**DOI:** 10.3389/fvets.2025.1616209

**Published:** 2025-07-03

**Authors:** Sarunas Badaras, Vytaute Starkute, Ernestas Mockus, Modestas Ruzauskas, Dovile Klupsaite, Erika Mozuriene, Jurgita Dailidaviciene, Agila Dauksiene, Laurynas Vadopalas, Barbara U. Metzler-Zebeli, Elena Bartkiene

**Affiliations:** ^1^Institute of Animal Rearing Technologies, Faculty of Animal Sciences, Lithuanian University of Health Sciences, Kaunas, Lithuania; ^2^Department of Food Safety and Quality, Faculty of Veterinary Medicine, Lithuanian University of Health Sciences, Kaunas, Lithuania; ^3^Department of Anatomy and Physiology, Faculty of Veterinary Medicine, Lithuanian University of Health Sciences, Kaunas, Lithuania; ^4^Institute of Microbiology and Virology, Faculty of Veterinary Medicine, Lithuanian University of Health Sciences, Kaunas, Lithuania; ^5^Centre for Veterinary Systems Transformation and Sustainability, Clinical Department for Farm Animals and Food System Science, University of Veterinary Medicine, Vienna, Austria

**Keywords:** faeces bacterial composition, immunoglobulins, faeces volatile compounds, antimicrobials, metataxonomic

## Abstract

Recently, fermented feed materials (FFM) have gained attention for their potential to improve overall performance in piglets. In this study, the effect of supplementing FFM to the diet of Topigs Norsvin Yorkshire piglets (weaning) on growth performance and health parameters was investigated. The whole experiment was divided into two phases: suckling (days 7 to 25) and weaning (days 25 to 69). During the suckling phase, 36 piglets (divided into three groups of 12 piglets/group) were assigned to three groups to differently ‘program’ their gut: (1) control (C) group, receiving a full-fledged commercial pre-starter feed, and (2) the Pp and (3) Pa groups, which received 25 mL of fermented milk permeate prepared either with *Pediococcus pentosaceus* LUHS183 and *Pediococcus acidilactici* LUHS29, respectively. In weaning, the pigs received two diets: C group received a non-fermented basal diet; Pp and Pa—same *Lactiplantibacillus plantarum* LUHS122, *Lactobacillus casei* LUHS210, *Latilactobacillus curvatus* LUHS51, and *Lacticaseibacillus paracasei* LUHS244 FFM. Results showed that weaned pigs of the Pp and Pa groups had higher body weight on day 69 compared to C group. Feed conversion ratio was similar in all three groups. On day 69, the highest concentration of immunoglobulins IgG was found in Pa group compared to other groups, while plasma alanine aminotransferase (ALT) levels were lower in treated groups compared to the C group. Diet did not influence ALT, aspartate aminotransferase (AST), faecal pH or dry matter content. On day 69, the faeces of the Pp and Pa groups exhibited higher texture hardness compared to the control (C) group. Additionally, the lactic acid bacteria (LAB) count differed significantly between the Pa and control groups. The C group had high abundances of beneficial lactobacilli and *Prevotellaceae* but the lowest bacterial diversity compared to the Pp and Pa groups. On day 69, faeces of treated groups had greater variability in individual volatile compounds (VCs) compared to the C group. Significant correlations between VC and faecal microbiological parameters were observed. In conclusion, the findings from this study show that with pediococci (LUHS183 and LUHS29), and lactobacilli FFM supports gut microbial diversification and homeostasis, potentially leading to improved BW gain.

## 1 Introduction

The gut microbiome has long been recognized for its crucial role in animal health and overall well-being. In pigs, the early establishment and maintenance of a beneficial gut microbiota are essential, as the first microbial colonizers play a key role in shaping the long-term microbial community, which ultimately influences the health and growth performance of pigs as they mature (1). It has been reported that the maternal gut microbiota of sows plays a key role in regulating fetal metabolism and immune function throughout pregnancy (2). Given the importance of these early gut colonizers, it is vital to comprehend the factors (diet, antimicrobials, probiotics, and prebiotics, among others) that influence the development of the piglet microbiome at weaning. Each of these elements can affect the piglet’s gut microbiome at weaning, influencing microbial diversity, structure, and succession within the gut. The gut microbiota offers the pig numerous benefits, including improved energy harvesting, production of volatile fatty acids, synthesis of vitamin K, cellulose fermentation, and enhanced resistance to pathogenic bacteria (3–5). As this field of study continues to evolve, it is clear that new roles and interactions between the gut microbiome and animal growth performance are still to be discovered. Additionally, further research is needed to investigate how supplementing FFM could impact piglet growth and health, particularly during the weaning period and in later stages of life. Weaning induces physiological changes in the structure and function of the intestine (6). Moreover, the gut microbiota of young piglets undergoes rapid ecological succession in response to various factors during the weaning period. One of the key factors is the abrupt change in diet from simple to more complex nutrient sources, which can affect the absorption capacity of the small intestine and likely influence growth and feed efficiency. During the weaning period piglets are exposed to thousands of new bacterial species, which may play significant roles in establishing an adult-like microbiota later in life. Additionally, establishing a beneficial microbiota during weaning is crucial, as piglets still have an immature immune system and rely on sow’s milk to prevent the colonization and overgrowth of opportunistic pathogens.

A study by Badaras et al. (7) reported that supplementing Topigs Norsvin Yorkshire newborn piglets with milk permeate fermented by lactic acid bacteria (LAB) strains *Pediococcus pentosaceus* LUHS183 (MPPp) and *P. acidilactici* LUHS29 (MPPa), which possess antimicrobial properties, could lead to beneficial changes in piglet growth performance and health parameters (7). The study concluded that feeding piglets with MPPa was beneficial for promoting weight gain, as well as fostering the growth of specific bacterial species and contributing to a distinct volatile compound (VC) profile in their faeces. While piglets fed with MPPp showed the highest IgM concentration in blood plasma. Both experimental groups demonstrated increased *Lactobacillus* counts in the faeces. The novelty of this study lies in the use of a novel combination of *Lactiplantibacillus plantarum* LUHS122, *Lactobacillus casei* LUHS210 *Latilactobacillus curvatus* LUHS51, and *Lacticaseibacillus paracasei* LUHS244 strains, which possess antimicrobial properties (8), for the preparation of fermented feed material (FFM), and in testing its influence on piglets, previously fed fermented milk permeate during the suckling period (7), throughout the weaning period.

The whole experiment was divided into two phases: suckling and weaning. During the suckling phase (from day 7 to 25 of life), 36 Topigs Norsvin Yorkshire piglets (divided into three groups of 12 piglets/group) were assigned to (1) the control (C) group, receiving a full-fledged commercial pre-starter feed (FFCP), and (2) the Pp and (3) Pa groups, receiving 25 mL of fermented milk permeate with *Pediococcus pentosaceus* LUHS183 and *Pediococcus acidilactici* LUHS29, respectively. During the suckling phase, the piglets had access to the sow’s milk till day 25 (7). The experiment began at the weaning phase (from the 25th day of life) and lasted for 43 days. After, we hypothesize that continuing feeding of piglets (from suckling to weaning and in the later stages) with FFM may be beneficial for enhancing their growth performance and health parameters. Therefore, in this study, the effect of supplementing FFM to the diet of piglets weaning on growth performance and health parameters including body weight (BW), feed conversion ratio (FCR), plasma immunoglobulins (IgA, IgM, IgG), aspartate aminotransferase (AST) and alanine aminotransferase (ALT) concentrations, faecal microbiological parameters, pH, dry matter (DM), metataxonomic and the faecal volatile compound (VC) profiles were investigated.

## 2 Materials and methods

### 2.1 Fermented feed preparation

The *Lb. plantarum* LUHS122, *Lb. casei* LUHS210, *Lb. curvatus* LUHS51, and *Lb. paracasei* LUHS244 strains were obtained from the Lithuanian University of Health Sciences collection (Kaunas, Lithuania). Our previous studies have shown that the aforementioned strains inhibit various pathogenic and opportunistic microorganisms (8). Characteristics of the LAB strains used are given in Supplementary File 1. The LAB strains were stored at −80°C in a Microbank system (Pro-Lab Diagnostics, UK) and separately propagated in de Man-Rogosa-Sharpe (MRS) broth (CM 0359, Oxoid Ltd., Hampshire, UK) at 30 ± 3°C for 48 h before their use for feed fermentation.

**Figure 1:**
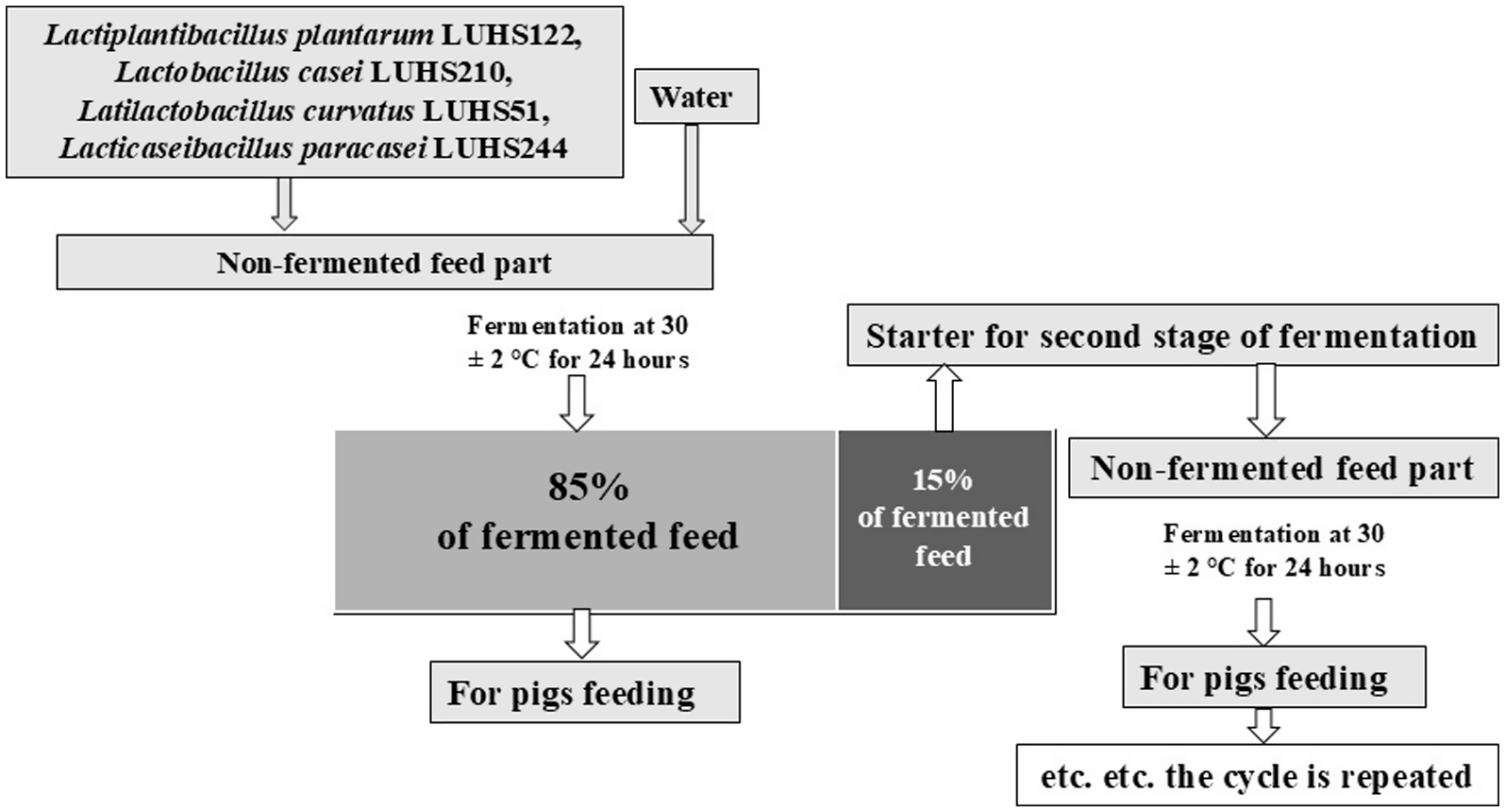
The principal scheme of the experiment of feed fermentation.

The fermented feed part (composition: crude protein—20.08%, crude fiber—7.34%, crude oil and fats—6.23%, lysine—1.01%, methionine—0.40%, tryptophan—0.28%, threonine—0.87%, Ca—0.33%, total P—0.64%, and Na—0.02%, NaCL—0.11%, Mg—0.27%, K—0.76%, S—0.2%, starch—31.95%, sugar—5.9%), water, and LAB strains (equal parts of each strain by volume) suspension (3% from dry matter of feed mass, v/m), containing 8.9 log_10_ CFU/mL, was fermented at 30 ± 2°C for 24 h. The final moisture content of the feed was 60 g/100 g. The moisture content was determined by drying the samples at 103 ± 2°C to a constant weight. Whole fermented feed mass (100%) was divided into two parts (15 and 85%, by mass): 85% of the fermented feed was used for piglet feeding, and 15% was used as a starter for additional feed fermentation cycles. The principal scheme of the experiment of feed fermentation is shown in Figure 1. To ensure the stability of the quality of each batch, a WEDA (Dammann & Westerkamp GmbH, Germany) feeding system was used for animal feeding. The system is equipped with integrated industrial pH meters and thermometers. An alarm is triggered if the temperature or pH reaches the specified limit values.

### 2.2 Animals’ housing and experimental design

All animal procedures were conducted according to the EU Directive (9) of the European Parliament and of Council from 22 September 2010 on the protection of animals used for scientific purposes and Requirements for the Keeping, Maintenance and Use of Animals Intended for Science and Education Purposes, approved by the order of the Lithuanian Director of the State Food and Veterinary Service (10).

The study was conducted at a pig farm in the Klaipeda district (Kantvainų vill., Lithuania) and at the Institute of Animal Rearing Technologies, Lithuanian University of Health Sciences (Kaunas, Lithuania). The farm participates in the Lithuanian Agricultural and Rural Development 2023–2027 of the intervention measure “Animal welfare” of the Strategic Plan, the activity “Support for 20%” “Ensuring a larger housing area for fattening pigs” implementation rules were prepared during the implementation of the 2021 December 2 Regulation (EU) 2021/2115 of the European Parliament and of the Council (11). Piglets are also raised with unclipped tails and unground.

**Figure 2:**
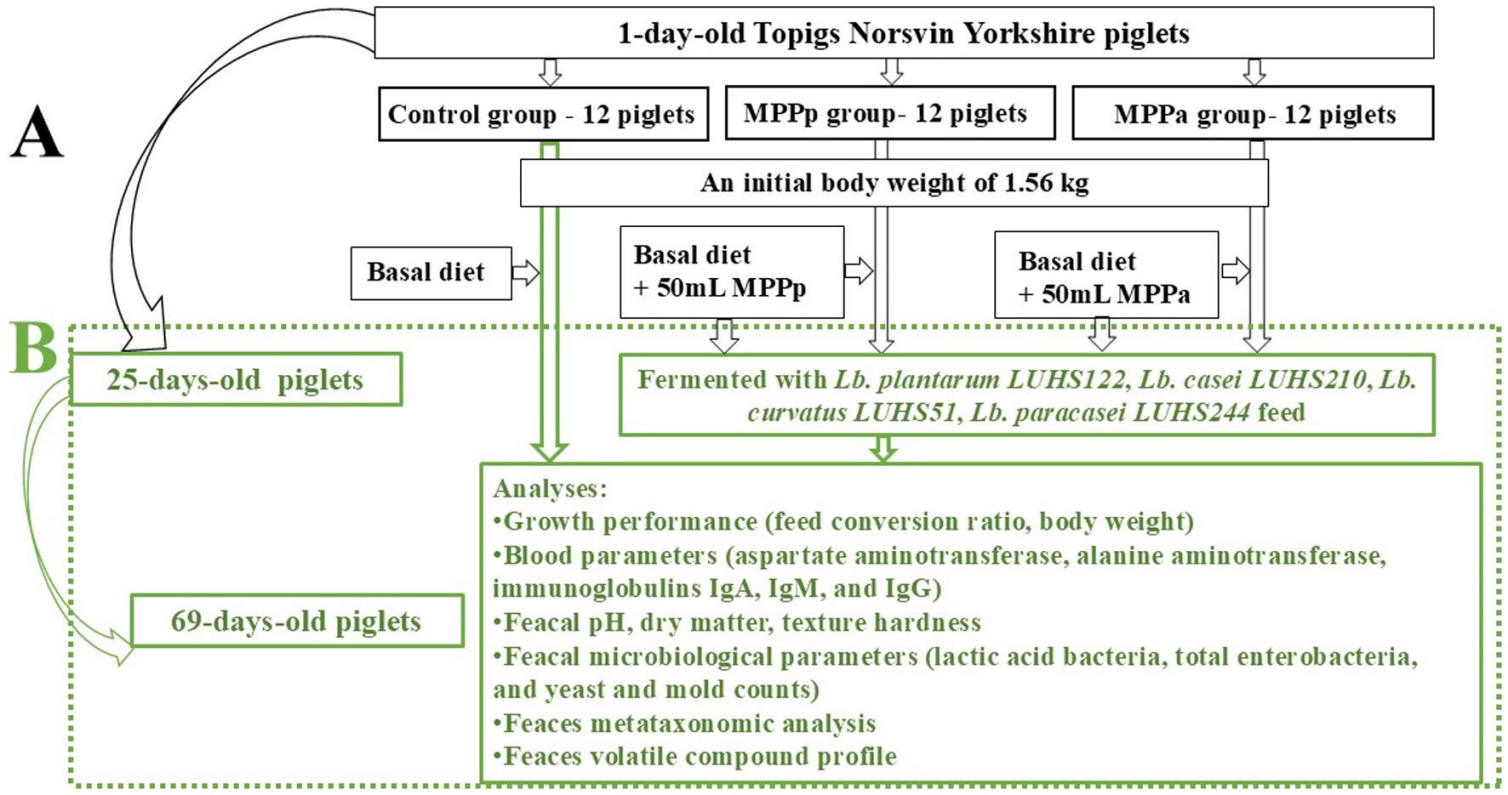
The principal scheme of the experiment (Part A: C, control group—fed with basal diet; MPPp, treated piglets group fed with basal diet and with 25 mL/day fermented with *Pediococcus pentosaceus* LUHS183 milk permeate; MPPa, treated piglets group fed with basal diet and with 25 mL/day fermented with *Pediococcus acidilactici* LUHS29 (2); Part B: continuous experiment: two dietary treatments for three piglet groups were compared (C, control group—fed with basal non-fermented diet, previously received full-fledged combined pre starter feed for piglets PANTO^®^; for treated groups, which additionally to traditional diet from the 7th day of life received fermented with Pp and Pa milk permeate (groups Pp and Pa, respectively) during the continuous experiment additionally received fermented with *Lb. plantarum* LUHS122, *Lb. casei* LUHS210, *Lb. curvatus* LUHS51, and *Lb. paracasei* LUHS244 feed material)).

The principal scheme of the whole experiment is shown in Figure 2.

The whole experiment was divided into two phases: suckling (days 7 to 25) and weaning (days 25 to 69). During the suckling phase (from day 7 to 25 of life), 36 Topigs Norsvin Yorkshire piglets (divided into three groups of 12 piglets/group) were assigned to (1) the control (C) group, receiving a full-fledged commercial pre-starter feed (FFCP), and (2) the Pp and (3) Pa groups, receiving 25 mL of fermented milk permeate with *P. pentosaceus* LUHS183 and *P. acidilactici* LUHS29, respectively. During the suckling phase, the piglets had access to the sow’s milk till day 25. Results of the suckling phase are reported by Badaras et al. (7).

The experiment began at the weaning phase (from the 25th day of life) and lasted for 43 days. At weaning, the body weight of the piglets was 6.95 ± 0.07 kg in the control (C), Pp and Pa groups. The diet of piglets (from 25th till 69th days) was composed of crude protein—19.09%, crude fiber—3.01%, crude fats—5.98%, av. lysine—1.55%, av. methionine—0.67%, av. tryptophan—0.25%, av. threonine—0.98%, Ca—0.86% and total P—0.62%. The weaned pigs were kept in a section with two climate zones. The first had a heated concrete floor (32°C) with a roof over it, while the second had plastic piglet floors and optimally ventilated air and temperature for the active period. The barn also housed other piglets from the same weaning week. It contained 24 pens—12 on the right and 12 on the left—separated by a central drive path. The feeding system was designed to deliver two different feed formulations through a single feeding tube within the same barn: fermented feed to the left side and commercial feed to the right side. Both experimental groups were fed from the same trough, while the control group was fed from a separate trough to allow accurate calculation of feed consumption. Drinking water and compound liquid feed were available *ad libitum* throughout the trial. Antimicrobial treatment was not applied.

The piglets were distributed into three groups, and blood plasma samples from 6 animals per group were collected. Weaning, two dietary treatments were compared for three groups: (i) non fermented basal diet for piglets previously fed with full-fledged combined pre starter feed for piglets PANTO^®^ (control group) and (ii) fermented with *Lb. plantarum* LUHS122, *Lb. casei* LUHS210, *Lb. curvatus* LUHS51 and *Lb. paracasei* LUHS244 strains combination for piglets previously fed with fermented with Pp and Pa milk permeate (groups Pp and Pa, respectively) (Figure 2). Fermented feed comprised 450 g/kg of total feed. The basal feed was formulated according to the nutritional requirements prescribed in the Nutrient Requirements of Swine (12). The feed composition and nutritional values are shown in Table 1. Dietary components were selected according to the AOAC recommendations (13).

**Table 1:** Diet composition.

Ingredients (%)	Control group to 15 kg	Control group to 15–30 kg	Treated groups (Pp and Pa) to 15 kg	Treated groups (Pp and Pa) to 15–30 kg
	KOM-50*	KOM-51*	PRO-1*	PRO-2*
24 h Fermented wheat			22.5	22.5
24 h Fermented rapeseed meal			22.5	22.5
Barley	20.0	44.70	15.00	24.13
Wheat	22.66	21.00	11.10	17.49
Hulled soybean meal		19.00		
Maize	15.00			
Soybean meal	2.00		2.20	5.35
Potato protein	5.00		2.00	
Extruded rapeseed mix		5.00		
Sweet whey powder	8.00			
Sunflower oil		2.00		
Barley malt sprouts		3.00		
Corn gluten	3.00			
Nordic Soya concentrate	5.90		1.22	
Sugar beet pomace		2.00		
Sugar beet pulp	4.00		2.00	
Dextrose Monohydrate	6.50		2.00	1.00
Vegetable oil	2.90		3.65	3.47
Premix, amino acids and other additives, %	5.04	3.30	15.83	3.56
*Nutritional value*				
ME swine (MJ/kg)	14.40	13.59	13.95	13.30
Crude protein	18.00	18.86	18.50	17.5
Digestible protein	16.92	16.11	16.47	15.47
Crude fat	5.29	4.63	5.51	5.79
Crude fibre	2.81	3.85	5.03	5.15
Crude ash	5.13	4.86	5.38	5.35
Lysine	1.42	1.19	1.48	1.41
Methionine	0.55	0.51	0.55	0.53
Threonine	0.95	0.81	1.00	0.95
Tryptophan	0.30	0.25	0.30	0.29
(Ca)—Calcium	0.85	0.69	0.80	0.75
(P)—Phosphorus (total)	0.51	0.42	0.66	0.63
(Na)—Sodium	0.22	0.15	0.16	0.18
NaCl	0.89	0.50	0.77	0.82
(Mg)—Magnesium	0.12	0.10	0.18	0.19
(K)—Potassium	0.64	0.57	0.57	0.63
(S)—Sulphur	0.18	0.16	0.17	0.17
Starch	34.10	40.16	38.23	39.02
Sugar	14.31	3.77	5.84	5.12

*KOM-50, KOM-51, PRO-1, PRO-2 are serial numbers of the feed production company AB “Kauno Grūdai” (Kaunas, Lithuania).

### 2.3 Evaluation of piglets’ growth performance

Both the experimental and control groups were weighed at age of 25, 26, 34, 41, 48, 55, 62, and 69 days of life using an electronic scale (model: IT1000, SysTec GmbH, Bergheim, Germany). To weigh only the selected piglets after driving them out of the barn, the necessary ones were separated in the corridor towards the scale, trying to cause the piglets as little stress as possible. All three study groups had individually numbered ear tags of different colors to facilitate their identification within the group. Feed conversion ratio (FCR) was calculated from feed intake (87% dry matter) and body weight (BW) gain recorded on the same days using a WEDA (Dammann & Westerkamp GmbH, Germany) automated feeding system. The feed components were mixed in a mixer and fed to pigs according to a feeding curve. The feed flow meter calculated the amount of feed added to each trough during feeding.

### 2.4 Blood plasma analysis

Blood samples were taken on days 25 (starting point) and 69 (end of FFM supplementation), before morning feeding. Piglets were fixed with a nose twister and blood was collected from the jugular vein into vacuum blood tubes (BD Vacutainer, Plymouth, UK) with a 18Gx1 ½″ needle number. The parameters included immunoglobulins IgA, IgM, IgG, aspartate aminotransferase (AST), and alanine aminotransferase (ALT) were analysed with an automatic biochemistry analyzer in the accredited laboratory “Anteja” (Klaipeda, Lithuania). Blood samples were collected by a licensed veterinarian (Sarunas Badaras veterinary practice license Nr. 1610) working on the farm.

### 2.5 Evaluation of faecal parameters

#### 2.5.1 Evaluation of faecal pH, dry matter, and texture hardness

For the evaluation of faecal pH, dry matter, texture hardness, and microbiological analysis, faecal samples were individually collected from 12 piglets in each group before (on day 25) and after (on day 69) the experiment. The samples were stored in vials at +4°C in a transport medium (Faecal Enteric Plus, Oxoid, Basingstoke, UK) and analysed on the same day.

#### 2.5.2 Microbiological analysis of faecal samples

For the evaluation of viable LAB count, 10 g of the faecal sample were homogenized with 90 mL of saline (9 g/L NaCl solution). Serial dilutions of 10^−4^ to 10^−8^ with saline were used for sample preparation. Sterile De Man, Rogosa and Sharpe (MRS) agar (CM0361, Oxoid) of 5 mm thickness was used for bacterial growth on Petri dishes. The dishes were separately seeded with the sample suspension using surface sowing and were incubated under anaerobic conditions at 30°C for 72 h. All results were expressed in log_10_ CFU/g (colony forming units per g of sample). Violet Red Bile Glucose (VRBG) agar (Oxoid Ltd., Basingstoke, United Kingdom) was used to analyze the total enterobacteria count (TEC) and Dichloran Rose Bengal Chloramphenicol (DRBC) agar (Liofilchem, Milan, Italy) was used to analyze yeast/mold (Y/M) count in faecal samples.

### 2.6 Metataxonomic analysis of the bacterial composition in the faecal samples of piglets’

On day 25 (starting point) and on day 69 (end of feed supplementation) faeces from all piglets in all the groups were collected using sterile cotton swabs. Pooled samples representing each of the group were made by mixing equal amount (0.5 g) of faeces taken individually, for targeted sequencing of 16S rRNA. Pooled samples were stored in were stored in DNA/RNA Shield (1:10 dilution; R1100-250, Zymo Research, USA) at −70°C until DNA extraction. ZymoBIOMICS^®^-96 MagBead DNA Kit (Zymo Research, Irvine, CA) was used to extract DNA using an automated platform according to manufacturers instructions. Bacterial 16S ribosomal RNA gene targeted sequencing was performed using the *Quick*-16S™ NGS Library Prep Kit (Zymo Research, Irvine, CA). The bacterial 16S custom-designed by Zymo Research primers amplified the V3-V4 region of the 16S rRNA gene. The sequencing library was prepared using real-time PCR. The final PCR products were quantified with qPCR fluorescence readings and pooled together based on equal molarity. The final pooled library was cleaned with the Select-a-Size DNA Clean & Concentrator™ (Zymo Research, Irvine, CA), then quantified with TapeStation^®^(Agilent Technologies, Santa Clara, CA) and Qubit^®^ (Thermo Fisher Scientific, Waltham, WA). The final library was sequenced on Illumina^®^ Nextseq™ with a P1 reagent kit (600 cycles). The sequencing was performed with 30% PhiX spike-in. Unique amplicon sequence variants were inferred from raw reads using the DADA2 pipeline (14). Potential sequencing errors and chimeric sequences were also removed with the Dada2 pipeline. Taxonomy assignment was performed using Uclust from Qiime v.1.9.1 with the Zymo Research Database, a 16S database that is internally designed and curated, as reference. Composition visualization and alpha-diversity analyse were performed with Qiime v.1.9.1 (15). The number of genome copies per microliter DNA sample was calculated by dividing the gene copy number by an assumed number of gene copies per genome.

### 2.7 Analysis of the faecal volatile compound profile

Faeces were prepared for gas chromatography (GC) analysis by using solid-phase microextraction (SPME). An SPME device with Stableflex (TM) fibre, coated with a 50-μm DVB-PDMS-Carboxen™ layer (Supelco, USA), was used for sample preparation. For gas chromatography–mass spectrometry (GC–MS), a GCMS-QP2010 (Shimadzu, Japan) was used. The gas chromatograph was equipped with an AOC-5000 Plus Shimadzu autosampler, upgraded with an SPME analysis kit. Analysis was performed according to the procedure described by Vadopalas et al. (16).

### 2.8 Statistical analysis

In order to compare the differences in parameter means between the different groups (C, Pp, Pa), a paired samples *t*-test was used (for growth performance (*n* = 12 for each group); for blood plasma parameters at the beginning and at the end of the experiment (*n* = 6 for each group); for the pH, dry matter, texture, VC profile, and microbiological parameters of faecal samples before (on day 25) and after (at day 69) the experiment (*n* = 12 for each group)). For metataxonomic analysis of metataxonomic analysis of the bacterial composition in the faecal samples, at the beginning of experiment, samples were collected from 12 piglets from each group and a single pooled sample was prepared for microbiome profiling, after the experiment, faeces from 12 piglets from the C, Pp, and Pa groups were collected and thereafter, three pooled samples, representing each of the groups, were prepared and analysed. The *p* values of factor (diet) influence were determined by multivariate tests of between-subjects effects. Baseline measurements were used as covariates to account for the experimental conditions. The mean values were compared using Duncan’s multiple range *post-hoc* test with the significance level defined at *p* ≤ 0.05. In the tables, the results are presented as mean values with pooled standard errors. Also, Pearson’s correlations between characteristics were calculated, strength of the correlations was interpreted according to Evans (17). Correlations were considered significant when *p ≤* 0.05. Differences in bacterial genera/species between the groups were assessed using the Z-test calculator for two population proportions (18). The number of reads of each genera/species from the total number of reads in samples was counted. Relative abundance of the genera/species in samples then were compared between the groups. Two-tailed hypothesis was counted. For the quality control of taxonomical identification, standard (D6300, Zymo Research, Murphy Ave. Irvine, CA, USA) of mixed known bacterial cultures were sequenced together with sample sequencing. Statistical comparisons were considered significant when *p* ≤ 0.05. Heatmap visualisation and analysis were conducted using the R statistical programming software package “ComplexHeatmap” (version 2.14.0). Partial least squares discriminant analysis (PLS-DA) and variable importance of projection (VIP) analyses were conducted using the “ropls” package (version 1.30.0). Volcano plots were generated with the “EnhancedVolcano” package (version 1.16.0). Missing values in samples (not detected in chromatogram) were filled using half minimum method. Pearson’s correlation analysis and visualizations were carried out using “psych” (version 2.4.12) and “corrplot” (version 0.95) packages.

## 3 Results

### 3.1 Piglets’ growth performance

**Figure 3:**
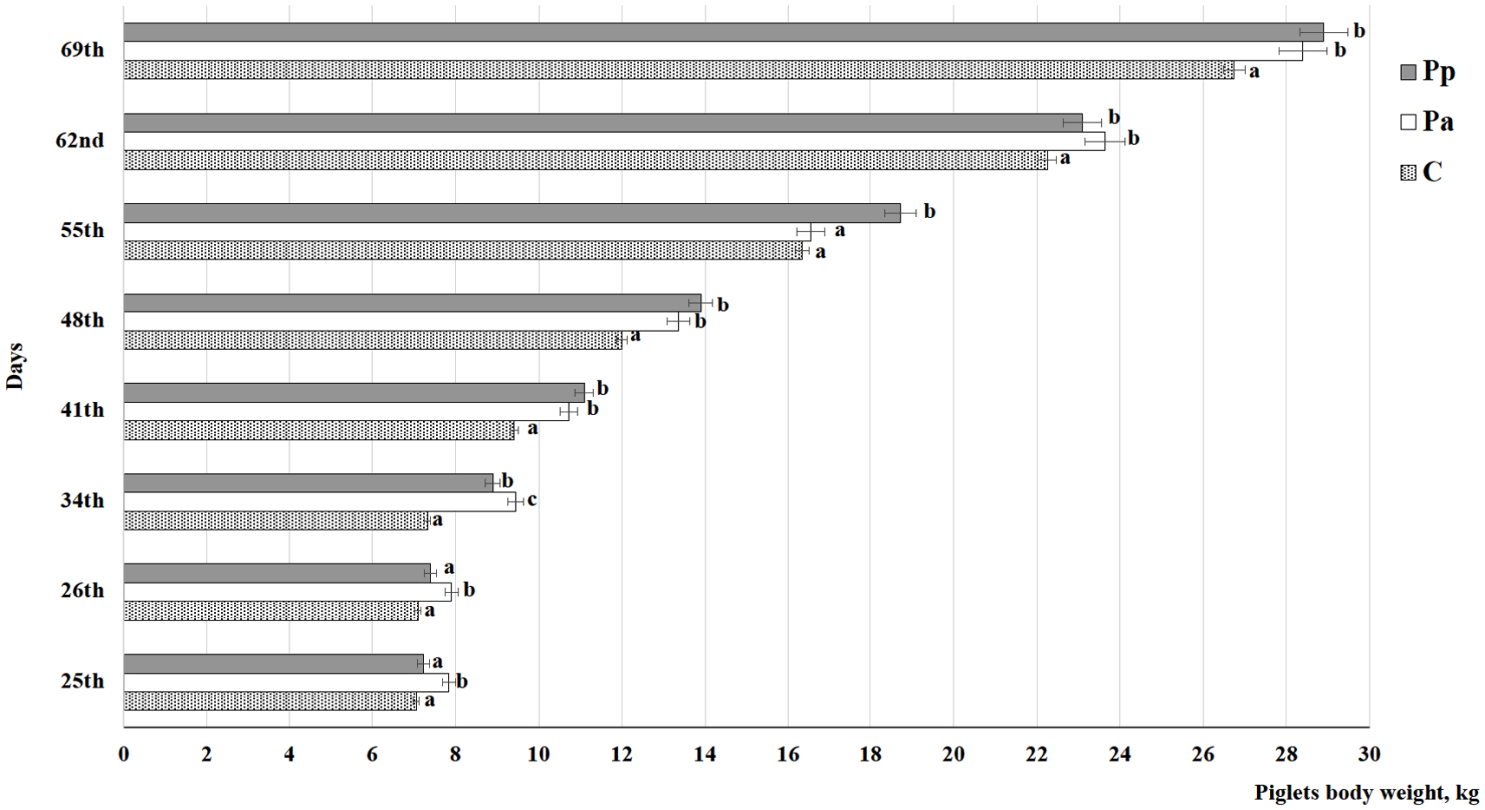
Piglets’ body weight, kg (C, control group—fed with basal non-fermented diet, previously received full-fledged combined pre starter feed for piglets PANTO^®^; Pp and Pa groups—previously, additionally to traditional diet from the 7th day of life received fermented with Pp and Pa milk permeate (groups Pp and Pa, respectively) and during the continuous experiment additionally received fermented with *Lb. plantarum* LUHS122, *Lb. casei* LUHS210, *Lb. curvatus* LUHS51, and *Lb. paracasei* LUHS244 feed material); ^a,c^Different letters indicate differences among different piglet groups on the same day (*p* ≤ 0.05). The data are presented as the mean ± standard deviation (*n* = 12/group).

Piglets’ body weights (kg) at age of 25, 26, 34, 41, 48, 55, 62, and 69 days of life are shown in Figure 3. On days 25 and 26, the piglets fed with Pa fermented milk permeate during the suckling phase had the highest body weight compared to the piglets fed the C and Pp fermented milk permeate (*p* < 0.05). On day 25, their body weight was, on average, 9.65% higher than that of the control group and the group previously fed with Pp fermented milk permeate. On day 26, their weight was, on average, 8.97% higher than that of the control group and the Pp group, respectively. However, on day 34, Pp piglets started to gain weight, and by day 41, both the Pa and Pp groups had, on average, 13.7% higher body weight than the control group. Similar trends were observed until the end of the experiment, except on day 55, when the Pa piglets’ body weight was lower compared to the Pp group.

**Figure 4:**
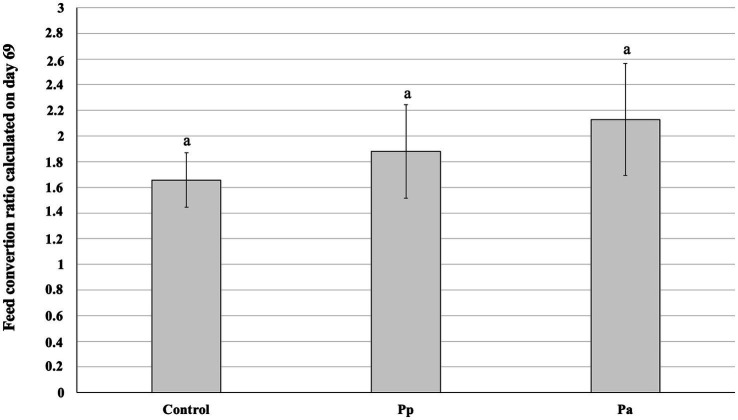
Feed conversion ratio calculated on day 69 (Control, control group—fed with basal non-fermented diet, previously received full-fledged combined pre starter feed for piglets PANTO^®^; Pp and Pa groups—previously, additionally to traditional diet from the 7th day of life received fermented with Pp and Pa milk permeate (groups Pp and Pa, respectively) and during the continuous experiment additionally received fermented with *Lb. plantarum* LUHS122, *Lb. casei* LUHS210, *Lb. curvatus* LUHS51, and *Lb. paracasei* LUHS244 feed material). The data are presented as the mean ± standard deviation (*n* = 12/group). ^a^ Different letters indicate differences among different piglet groups (*p* ≤ 0.05).

The feed conversion ratio (FCR) calculated on day 69 is shown in Figure 4. Significant differences in the FCR between the C, Pp, and Pa groups were not established.

### 3.2 Piglets’ blood plasma parameters

The blood plasma parameters of piglets on 25 and 69 days old are shown in Table 2. In all groups, at both ages, the IgA concentration in piglet blood plasma was <0.33 g/L. Although no significant differences were found between the piglet groups in IgM concentration in blood plasma, diet at 25 days was a significant factor for IgM levels (*p* = 0.003). Additionally, when comparing IgM concentration in piglet blood plasma on 25 and 69 days, higher IgM concentrations were found in the C and Pa groups on 69 days. Higher concentration of IgG was found in the blood plasma of Pa piglets on day 69, in comparison with the C group. However, no significant differences in IgG concentrations were found between the control and Pp groups, and diet was not a significant factor for IgG concentration. When comparing ALT concentration in piglet blood plasma, ALT levels on 69 days were lower in both treated groups (Pp and Pa) compared to the C group (*p* < 0.05). However, diet was not found to be a significant factor affecting ALT concentration. AST concentration was higher in the C and Pa groups versus the Pp group after 69 days of the experiment, but not on day 25. However, diet was not found to be a significant factor for AST levels in piglet blood plasma.

**Table 2:** Blood plasma parameters of 25- and 69-day-old piglets.

Blood parameters		Treatments			*p*			
		C	Pp	Pa	C × Pp	C × Pa	Pp × Pa	Significance of the analysed factor (diet)
Immunoglobulin (IgA), g/L	25th day	<0.330	<0.330	<0.330	–	–	–	–
	69th day	<0.330	<0.330	<0.330	–	–	–	–
Immunoglobulin (IgM), g/L	25th day	0.225 ± 0.028^a^	0.380 ± 0.144^a^	0.285 ± 0.086^a^	0.147	0.215	0.105	**0.003**
	69th day	0.578 ± 0.106^b^	0.556 ± 0.228^a^	0.568 ± 0.183^b^	0.784	0.843	0.690	0.896
Immunoglobulin (IgG), g/L	25th day	2.11 ± 0.281^a^	2.03 ± 0.447^a^	1.78 ± 0.613^a^	0.492	0.227	0.121	0.837
	69th day	2.61 ± 0.731^a^	2.13 ± 0.427^b^	3.52 ± 0.989^b^	0.112	**0.026**	**0.050**	0.758
ALT, U/L	25th day	52.0 ± 12.3^a^	46.7 ± 6.06^a^	44.2 ± 7.11^a^	0.279	0.121	0.054	0.376
	69th day	91.0 ± 14.4^b^	71.8 ± 16.3^a^	61.0 ± 14.3^a^	**0.003**	**<0.001**	**0.011**	0.051
AST, U/L	25th day	31.7 ± 10.6^a^	35.2 ± 6.05^a^	30.7 ± 4.68^a^	0.314	0.797	**0.030**	0.426
	69th day	40.2 ± 7.43^b^	39.5 ± 8.34^a^	33.0 ± 5.26^b^	0.314	**0.029**	0.067	0.466

C, control group—fed with basal non-fermented diet, previously received full-fledged combined pre starter feed for piglets PANTO^®^; Pp and Pa groups—previously, additionally to traditional diet from the 7th day of life received fermented with Pp and Pa milk permeate (groups Pp and Pa, respectively) and during the continuous experiment additionally received fermented with *Lb. plantarum* LUHS122, *Lb. casei* LUHS210, *Lb. curvatus* LUHS51, and *Lb. paracasei* LUHS244 feed material; 25th—at the beginning of the experiment; 69th—at the end of the experiment; ALT, alanine aminotransferase; AST, aspartate aminotransferase; ^a,b^Different letters indicate significant time-related differences (*p* ≤ 0.05). The data are presented as the mean ± standard deviation (*n* = 6/group). Bold values indicate significant difference between tested treatments or significant effect of the analyzed factor on tested parameters, when *p* ≤ 0.05.

### 3.3 Piglets’ faecal pH, dry matter, and texture hardness

The pH, dry matter, and texture hardness of piglets’ faeces are shown in Table 3. No significant differences in faecal pH were found between groups. However, on day 69, the highest faecal dry matter content was observed in the Pp piglets. Diet was not a significant factor for faeces pH or dry matter content. At the end of the experiment, both treated groups showed higher faecal texture hardness compared to the control group. However, on day 69, diet was not a significant factor for faecal texture hardness.

**Table 3:** The pH, dry matter and texture hardness of piglet faeces.

Faecal parameters		C	Pp	Pa	*p*			
					C × Pp	C × Pa	Pp × Pa	Significance of the analysed factor (diet)
pH	25th day	6.38 ± 0.75^a^	6.35 ± 0.85^a^	6.47 ± 0.78^a^	0.655	0.035	0.097	0.982
	69th day	6.45 ± 0.68^a^	6.04 ± 0.17^a^	6.12 ± 0.22^a^	0.298	0.340	0.109	0.226
Dry matter (%)	25th day	33.6 ± 6.76^b^	46.5 ± 12.6^b^	44.36 ± 14.6^b^	0.062	0.141	0.205	0.413
	69th day	30.6 ± 7.94^a^	36.5 ± 6.52^a^	31.4 ± 4.52^a^	**0.019**	0.725	**0.048**	0.486
Texture hardness (mJ)	25th day	0.420 ± 0.070^a^	0.210 ± 0.030^a^	0.210 ± 0.040^a^	**0.012**	**0.012**	1.00	**0.003**
	69th day	0.417 ± 0.072^a^	0.486 ± 0.122^b^	0.456 ± 0.151^b^	0.139	0.166	0.500	0.163

C, control group—fed with basal non-fermented diet, previously received full-fledged combined pre starter feed for piglets PANTO^®^; Pp and Pa groups—previously, additionally to traditional diet from the 7th day of life received fermented with Pp and Pa milk permeate (groups Pp and Pa, respectively) and during the continuous experiment additionally received fermented with *Lb. plantarum* LUHS122, *Lb. casei* LUHS210, *Lb. curvatus* LUHS51, and *Lb. paracasei* LUHS244 feed material; 25th—at the beginning of the experiment; 69th—at the end of the experiment; AST, aspartate aminotransferase; ALT, alanine aminotransferase; 25th, at the beginning of the experiment; 69th, at the end of the experiment; ^a,b^Different letters indicate significant time-related differences (*p* ≤ 0.05). The data are presented as the mean ± standard deviation (*n* = 12/group). Bold values indicate significant difference between tested treatments or significant effect of the analyzed factor on tested parameters, when *p* ≤ 0.05.

### 3.4 Microbiological parameters of piglets’ faeces

Microbiological parameters of piglet faecal samples are shown in Table 4. In all cases, a significantly higher total enterobacteria count (TEC) was observed in piglet faeces at day 25 of life. On day 69, no significant differences in TEC were found between the different groups. On day 69, the significantly higher LAB count was observed in the Pa group, compared to the control group (*p* = 0.026). However, no significant differences in LAB counts were found between the Pp and C group as well as between Pp and Pa groups. Additionally, the control group exhibited the highest yeast and mold counts (at the beginning and at the end of the experiment). In comparison, the yeast and mold count in the Pp and Pa groups were lower, with the Pp group showing the lowest count. At the end of the experiment, diet was a significant factor in the yeast and mold counts in piglet faeces.

**Table 4:** Microbiological parameters of piglets’ faecal samples.

Parameter	Day	C	Pp	Pa	*p*			
					C × Pp	C × Pa	Pp × Pa	Significance of the analysed factor (diet)
		Microorganisms count, log_10_ CFU/g						
C	25th day	6.14 ± 0.14^b^	7.13 ± 0.24^b^	7.41 ± 0.31^b^	**0.003**	**0.006**	**0.020**	**0.002**
	69th day	5.20 ± 0.34^a^	5.10 ± 0.46^a^	5.20 ± 0.33^a^	0.286	1.000	0.314	0.934
LAB	25th day	5.11 ± 0.35^b^	8.13 ± 0.25^b^	7.30 ± 0.41^b^	**<0.001**	**0.002**	**0.012**	**<0.001**
	69th day	4.17 ± 0.48^a^	3.95 ± 0.76^a^	4.24 ± 0.50^b^	0.307	**0.026**	0.193	0.828
Y/M	25th day	5.21 ± 0.21^b^	3.05 ± 0.08^a^	3.92 ± 0.10^b^	**0.012**	**0.002**	**<0.001**	**<0.001**
	69th day	4.22 ± 0.50^a^	1.14 ± 1.12^a^	2.33 ± 0.68^a^	**0.013**	**0.003**	**0.043**	**0.010**

TEC, total enterobacteria count; LAB, lactic acid bacteria; Y/M, yeast and mold; C, control group—fed with basal non-fermented diet, previously received full-fledged combined pre starter feed for piglets PANTO^®^; Pp and Pa groups—previously, additionally to traditional diet from the 7th day of life received fermented with Pp and Pa milk permeate (groups Pp and Pa, respectively) and during the continuous experiment additionally received fermented with *Lb. plantarum LUHS122*, *Lb. casei LUHS210*, *Lb. curvatus LUHS51* and *Lb. paracasei LUHS244* feed material; 25th—at the beginning of the experiment; 69th—at the end of the experiment; ^a,b^Different letters indicate significant time-related differences (*p* ≤ 0.05). The data are presented as the mean ± standard deviation (*n* = 12/group). Bold values indicate significant difference between tested treatments or significant effect of the analyzed factor on tested parameters, when *p* ≤ 0.05.

### 3.5 Metataxonomic composition of faecal microbiota

Table 5 demonstrates faecal bacterial composition in pigs’ faeces at a genus level on the 25th day.

**Table 5:** Differences in relative abundances (% of all reads) of bacterial genera in pigs’ faeces on the 25th day.

Taxonomy*	Group			*p*		
	C	Pa	Pp	C × Pp	C × Pa	Pp × Pa
*Methanobrevibacter*	3.9%	3.2%	1.7%	**0.003**	0.395	**0.03**
*Bacteroides*	5.1%	3.8%	12.8%	**<0.001**	0.159	**<0.001**
*Bacteroidales*	2.9%	4.0%	4.6%	**0.05**	0.18	0.5
*Parabacteroides*	0.5%	1.8%	0.9%	0.285	**0.006**	0.08
*Prevotellaceae*	1.7%	2.9%	3.0%	0.054	0.073	0.896
*Rikenellaceae*	1.5%	1.8%	2.5%	0.109	0.596	0.280
*Enterococcus*	0.3%	0.0%	1.5%	**0.005**	0.083	**<0.001**
*Lactobacillus*	1.2%	2.3%	6.8%	**<0.001**	0.060	**<0.001**
*Christensenellaceae*	10.8%	10.6%	2.4%	**<0.001**	0.888	**<0.001**
*Clostridium*	4.9%	3.5%	2.9%	**0.021**	0.119	**0.045**
*Clostridiales I*	1.0%	1.1%	1.7%	0.174	0.826	0.254
*Blautia*	1.4%	1.6%	1.3%	0.849	0.711	0.575
*Lachnoclostridium*	2.4%	1.3%	3.2%	0.28	0.672	**0.004**
*Marvinbryantia*	2.4%	3.3%	0.6%	**<0.001**	0.226	**<0.001**
*Lachnospiraceae*	6.4%	8.2%	3.4%	**0.002**	0.121	**<0.001**
*Clostridiales II*	2.2%	5.1%	1.7%	0.418	0.001	**<0.001**
*Romboutsia*	2.5%	3.5%	4.0%	0.059	0.19	0.555
*Terrisporobacter*	1.6%	2.4%	0.4%	**0.007**	0.2	**<0.001**
*Anaerotruncus*	0.7%	0.9%	1.2%	0.25	0.617	0.509
*Ruminococcaceae*	21.0%	15.3%	14.9%	**<0.001**	**<0.001**	0.803
*Holdemanella*	0.4%	1.1%	0.8%	0.246	0.07	0.49
*Erysipelotrichaceae*	2.3%	2.1%	4.1%	**0.022**	0.764	**0.001**
*Turicibacter*	0.6%	1.1%	0.2%	0.156	0.222	**0.012**
*Phascolarctobacterium*	1.9%	1.3%	1.1%	0.142	0.285	0.682
*Fusobacterium*	0.0%	0.6%	1.9%	**<0.001**	**0.014**	**0.009**
*Escherichia*	3.1%	1.6%	3.1%	1	**0.027**	**0.027**
*Cloacibacillus*	1.5%	0.6%	1.3%	0.70	**0.049**	0.11
*Pyramidobacter*	0.2%	0.2%	2.5%	**<0.001**	1	**<0.001**

*Higher taxonomic ranks are presented in case of undetermined genus level during identification. Only those genera are presented which prevalence was at least 1% from all bacterial count in any of the group (C, control group—fed with basal non-fermented diet, previously received full-fledged combined pre starter feed for piglets PANTO^®^; Pp and Pa groups—previously, additionally to traditional diet from the 7th day of life received fermented with Pp and Pa milk permeate (groups Pp and Pa, respectively) and during the continuous experiment additionally received fermented with *Lb. plantarum* LUHS122, *Lb. casei* LUHS210, *Lb. curvatus* LUHS51, and *Lb. paracasei* LUHS244 feed material). Bold values indicate significant difference between tested treatments or significant effect of the analyzed factor on tested parameters, when *p* ≤ 0.05.

**Figure 5:**
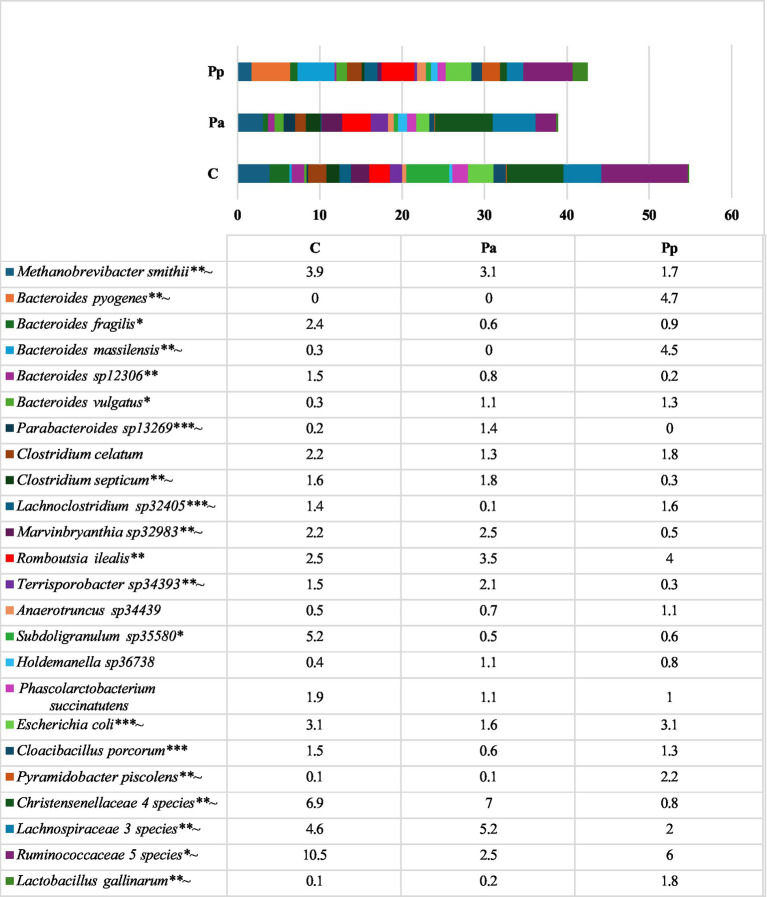
Differences in relative abundances (% of all reads) of bacterial amplicon sequence variants (ASV) in the pigs’ faeces on the 25th day (Only these ASV are presented which prevalence was ≥ 1% from all bacterial count in any of the animal group; C, control group—fed with basal non-fermented diet, previously received full-fledged combined pre starter feed for piglets PANTO^®^; Pp and Pa groups—previously, additionally to traditional diet from the 7th day of life received fermented with Pp and Pa milk permeate (groups Pp and Pa, respectively) and during the continuous experiment additionally received fermented with *Lb. plantarum* LUHS122, *Lb. casei* LUHS210, *Lb. curvatus* LUHS51, and *Lb. paracasei* LUHS244 feed material). *Statistically significant results (*p* ≤ 0.05) between the control and experimental groups; **Statistically significant results (*p* ≤ 0.05) between the control and Pp group; ***Statistically significant results (*p* ≤ 0.05) between the control and Pa group; ~Statistically significant results (*p* ≤ 0.05) between Pa and Pp groups.

On day 25, in all the groups of pigs, the most prevalent bacteria belonged to an unclassified genus within the family *Ruminococcaceae* with a relative abundance of 14.9 to 21.0% of all sequences. The second most prevalent family in the C and Pa groups was *Christensenellaceae*, with a relative abundance of 10.8 and 10.6%, respectively. In the Pp group, the second most prevalent genus was *Bacteroides*, with a relative abundance of 12.8%. Bacteria from this genus were also highly abundant in the other two groups. The most prevalent bacteria detected at species level on the 25th day are presented in Figure 5.

Data from amplicon sequencing showed a very complex bacterial community in pigs of all groups, without clearly dominant species however, the overall composition has some differences in each animal group. In the C group the most prevalent species were *Methanobrevibacter smithii*, *Escherichia coli*, *Bacteroides fragilis*, *Romboutsia ilealis*, *Clostridium celatum*, *Marvinbryanthia* sp32983 and different species from *Rumonococcaceae*, *Christensenellaceae* and *Lachnospiraceae*. Besides from those species, in group Pa the most abundant species were *Romboutsia ilealis*, *Methanobrevibacter smithii*, *Marvinbryanthia* sp32983 and *Terrisporobacter* sp34393, whereas in Pp group—*Bacteroides pyogenes*, *Bacteroides massilensis*, *Romboutsia ilealis*, *Escherichia coli* and *Pyramidobacter piscolens*. This group also comprised the highest relative abundance of *Lactobacillus gallinarum* (1.8%) and other *Lactobacillus* spp.

On day 69, the most prevalent genera/families in faeces of the C group were *Lactobacillus* (19.8%), *Prevotellaceae* (11.0%), *Ruminococcaceae* (10.1%) and *Lachnospiraceae* (7.4%) which made almost half (48.3%) of all the bacterial load in pigs’ faeces. In Pp group, the most prevalent genera/families were *Ruminococcaceae* (14.9%), *Prevotellaceae* (10.1%), *Lactobacillus* (5.7%) and *Lachnospiraceae* (5,5%), i.e., the same bacterial genera as in the control group. In Pa group the most prevalent genera/families were *Ruminococcaceae* (17.4%), *Lachnospiraceae* (10.2%), *Prevotellaceae* (7.7%), *Lactobacillus* (4.4%) and *Bacteroidales* (5.0%). *Bacteroidales* also were abundant in other two groups as well. Bacterial composition on the 69th day at the genus level in all the animal groups is presented in Table 6.

**Table 6:** Differences in relative abundances (% of all reads) of bacterial genera in pigs’ faeces on day 69.

Taxonomy*	Group			*p*		
	C	Pa	Pp	C × Pp	C × Pa	Pp × Pa
*Methanobrevibacter*	0.0%	0.4%	1.6%	**<0.001**	**0.046**	**0.007**
*Methanosphaera*	0.2%	1.3%	0.1%	0.562	**0.004**	**0.001**
*Bacteroidales*	2.1%	4.1%	5.0%	**<0.001**	**0.01**	0.332
*Alloprevotella*	2.0%	1.8%	1.6%	0.5	0.741	0.726
*Prevotellaceae*	11.0%	10.1%	7.7%	**0.011**	0.51	0.06
*Prevotella*	3.2%	4.0%	3.6%	0.624	0.337	0.638
*Rikenellaceae*	1.3%	2.0%	3.4%	**0.002**	0.219	0.536
*Lactobacillus*	19.8%	5.7%	4.2%	**<0.001**	**<0.001**	0.121
*Christensenellaceae*	0.9%	2.0%	3.3%	**<0.001**	**0.04**	0.07
*Clostridium*	0.8%	2.5%	1.2%	0.368	**0.003**	0.368
*Clostridiales; Family XIII*	0.5%	1.4%	1.2%	0.09	**0.004**	0.697
*Blautia*	3.5%	3.3%	3.9%	0.638	0.8	0.472
*Coprococcus*	1.6%	2.1%	2.3%	0.258	0.41	0.764
*Eubacterium*	0.6%	1.2%	0.8%	0.589	0.156	0.368
*Marvinbryantia*	1.0%	0.8%	1.1%	0.825	0.638	0.49
*Lachnospiraceae*	7.4%	5.5%	10.2%	**0.027**	0.084	**<0.001**
*Oribacterium*	1.1%	1.6%	1.1%	1	0.332	0.332
*Roseburia*	2.4%	2.1%	1.9%	0.441	0.653	0.749
*Clostridiales*	2.4%	2.7%	1.4%	0.101	0.667	**0.04**
*Romboutsia*	0.4%	2.1%	1.0%	0.107	**<0.001**	**0.046**
*Terrisporobacter*	3.2%	5.1%	1.8%	**0.044**	**0.033**	**<0.001**
*Faecalibacterium*	1.8%	0.9%	1.3%	0.363	0.082	0.39
*Ruminococcaceae*	10.1%	14.9%	17.4%	**<0.001**	**0.001**	0.129
*Holdemanella*	1.2%	0.4%	0.5%	0.087	**0.044**	0.741
*Erysipelotrichaceae*	1.2%	1.4%	1.6%	0.447	0.696	0.711
*Solobacterium*	0.9%	1.6%	1.8%	0.081	0.158	0.726
*Phascolarctobacterium*	2.1%	1.4%	1.9%	0.749	0.234	0.379
*Dialister*	1.0%	0.2%	0.3%	0.512	**0.02**	0.653
*Megasphaera*	0.1%	1.4%	0.2%	0.561	**<0.001**	**0.003**
*Mollicutes*	3.1%	1.7%	0.8%	**<0.001**	**0.04**	0.07
*Anaerovibrio*	0.2%	0.6%	1.1%	**0.012**	0.156	0.222

*Higher taxonomic ranks are presented in case of undetermined genus level during identification. Only those genera are presented which prevalence was at least 1% from all bacterial count in any of the group (C, control group—fed with basal non-fermented diet, previously received full-fledged combined pre starter feed for piglets PANTO^®^; Pp and Pa groups—previously, additionally to traditional diet from the 7th day of life received fermented with Pp and Pa milk permeate (groups Pp and Pa, respectively) and during the continuous experiment additionally received fermented with *Lb. plantarum*, *Lb. casei* LUHS210, *Lb. curvatus* LUHS51, and *Lb. paracasei* LUHS244 feed material). Bold values indicate significant difference between tested treatments or significant effect of the analyzed factor on tested parameters, when *p* ≤ 0.05.

**Figure 6:**
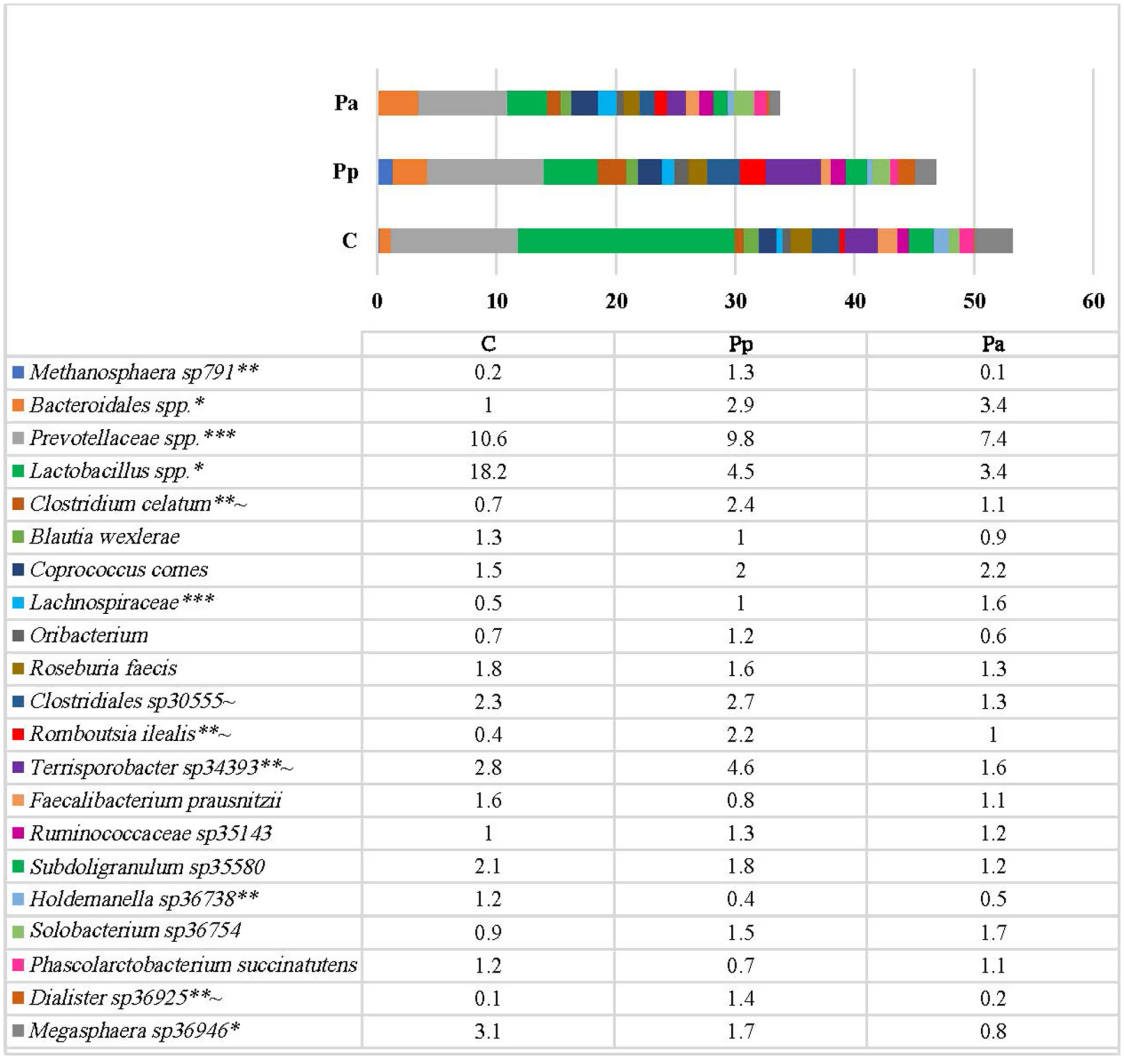
Differences in relative abundances (% of all reads) of bacterial amplicon sequence variants (ASV). Only these species are presented which prevalence was ≥ 1% from all bacterial count in any of the animal group (C, control group—fed with basal non-fermented diet, previously received full-fledged combined pre starter feed for piglets PANTO^®^; Pp and Pa groups—previously, additionally to traditional diet from the 7th day of life received fermented with Pp and Pa milk permeate (groups Pp and Pa, respectively) and during the continuous experiment additionally received fermented with *Lb. plantarum* LUHS122, *Lb. casei* LUHS210, *Lb. curvatus* LUHS51, and *Lb. paracasei* LUHS244 feed material). *Statistically significant results (*p* ≤ 0.05) between the control and experimental groups; **Statistically significant results (*p* ≤ 0.05) between the control and Pp group; ***Statistically significant results (*p* ≤ 0.05) between the control and Pa group; ~Statistically significant results (*p* ≤ 0.05) between Pa and Pp groups.

On the species level (Figure 6) the most prevalent bacteria in the control group were different species of *Lactobacillus*, from which only minority of them were identified up to the species level (*L. pontis*, *L. amylovorus*, *L. panis* species) which has the highest prevalence among all other bacteria (18.2%). The other most prevalent species in this group were different species of *Prevotellaceae* (10.6%), *Terrisporobacter* (2.8%) and *Clostridiales* (2.3%). In Pp group the most prevalent bacteria were species from *Prevotellaceae* (9.8%), *Lactobacillus* (4.5%), *Terrisporobacter* (4.6%), and *Bacteroidales* (2.9%). In group Pa, the most prevalent bacteria were *Prevotellaceae* (7.4%), *Lactobacillus* (3.4%) and *Bacteroidales* (3.4%).

**Figure 7:**
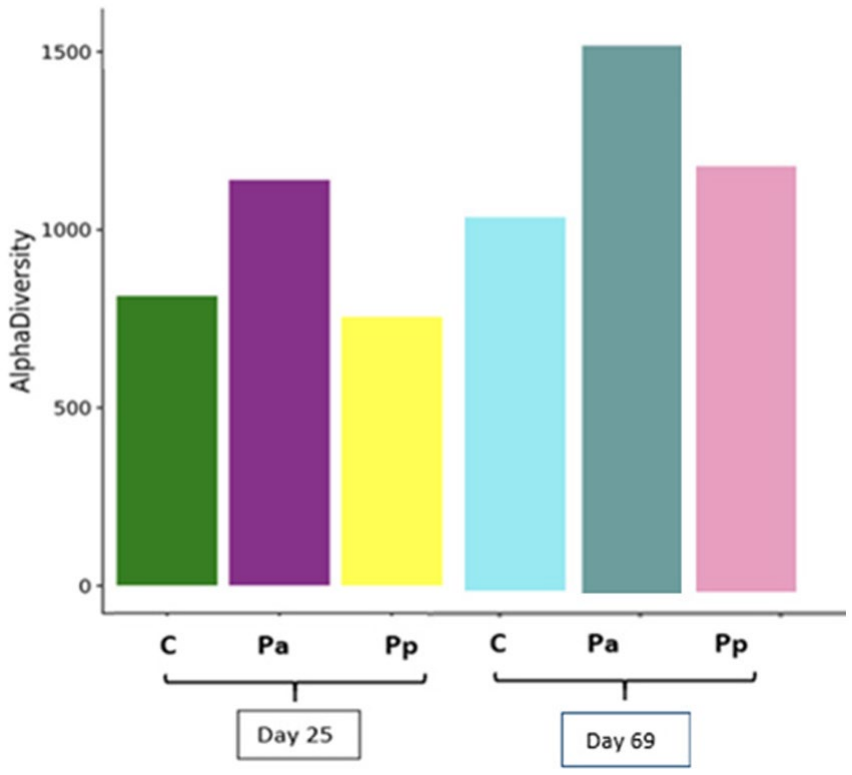
Species richness of the bacterial communities in the faeces of pigs on day 25 and 69 (C, control group—fed with basal non-fermented diet, previously received full-fledged combined pre starter feed for piglets PANTO^®^; Pp and Pa groups—previously, additionally to traditional diet from the 7th day of life received fermented with Pp and Pa milk permeate (groups Pp and Pa, respectively) and during the continuous experiment additionally received fermented with *Lb. plantarum* LUHS122, *Lb. casei* LUHS210, *Lb. curvatus* LUHS51, and *Lb. paracasei* LUHS244 feed material).

Figure 7 demonstrates alpha diversity (richness of the species) among all animal groups on days 25 and 69.

It was determined that species variety increased with the pig age. The higher richness of species diversity was detected at the end of the feeding trial in comparison with day 25. Although the C group has highest numbers of some beneficial bacteria (*Lactobacillus* and *Prevotellaceae*), it also has the lowest bacterial richness in comparison with experimental groups of pigs.

### 3.6 Volatile compound profiles of piglets’ faeces

**Figure 8:**
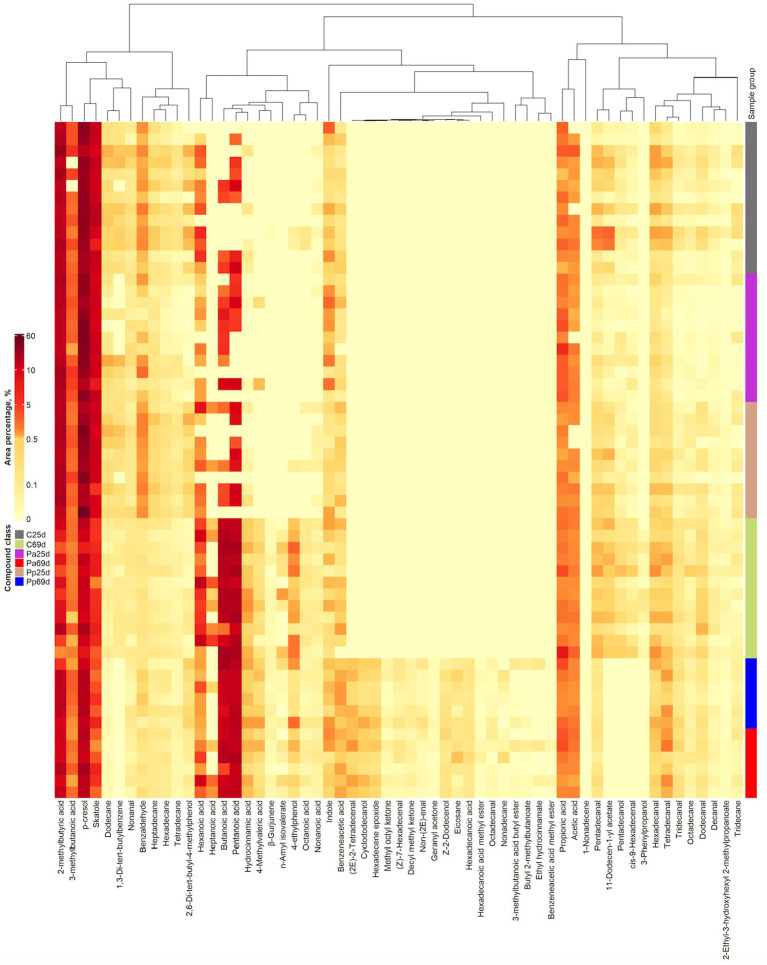
Volatile compound profiles of piglets’ faeces (C, control group—fed with basal non-fermented diet, previously received full-fledged combined pre starter feed for piglets PANTO^®^; Pp and Pa groups—previously, additionally to traditional diet from the 7th day of life received fermented with Pp and Pa milk permeate (groups Pp and Pa, respectively) and during the continuous experiment additionally received fermented with *Lb. plantarum* LUHS122, *Lb. casei* LUHS210, *Lb. curvatus* LUHS51, and *Lb. paracasei* LUHS244 feed material; 25th—at the beginning of the experiment; 69th—at the end of the experiment).

Volatile compound (VC) profiles of piglets’ faeces are shown in Figure 8. When comparing the faecal VC profiles on day 69 between C and treated groups, butyl 2-methylbutanoate, 3-methylbutanoic acid butyl ester, non-(2E)-enal, methyl octyl ketone, benzeneacetic acid methyl ester, ethyl hydrocinnamate, decyl methyl ketone, geranyl acetone, Z-2-dodecenol, cyclododecanol, (2E)-2-tetradecenal, hexadecene epoxide, (Z)-7-hexadecenal, nonadecane, hexadecanoic acid methyl ester, hexadecanoic acid, eicosane, and octadecanal were detected only in the faeces of the Pa and Pp groups but not in those of the C group. On the other hand, 1,3-di-tert-butylbenzene, 11-dodecen-1-yl acetate, pentadecanol, and cis-9-hexadecenal were found exclusively in the faeces to the C group.

**Figure 9:**
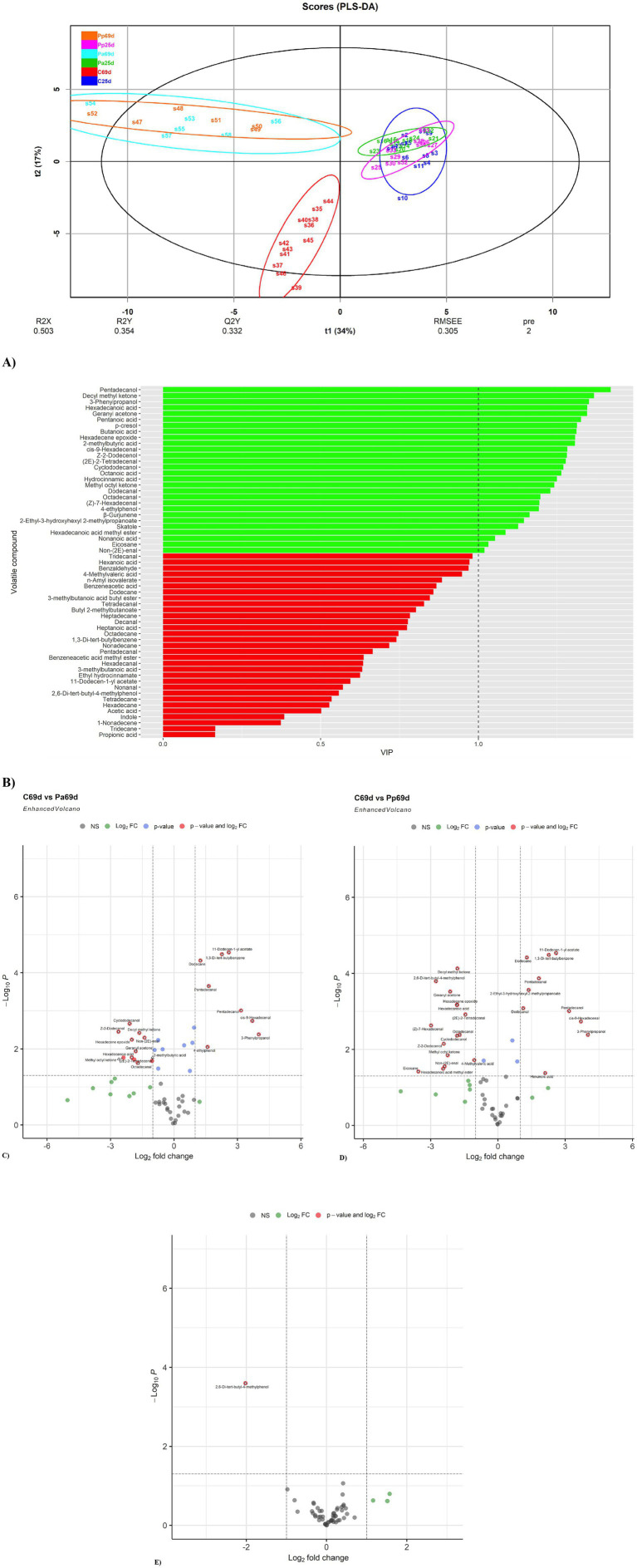
**(A)** PLS-DA plot of the detected VCs in piglet faecal sample groups (C, control group—fed with basal non-fermented diet, previously received full-fledged combined pre starter feed for piglets PANTO^®^; Pp and Pa groups—previously, additionally to traditional diet from the 7th day of life received fermented with Pp and Pa milk permeate (groups Pp and Pa, respectively) and during the continuous experiment additionally received fermented with *Lb. plantarum* LUHS122, *Lb. casei* LUHS210, *Lb. curvatus* LUHS51, and *Lb. paracasei* LUHS244 feed material; 25th—at the beginning of the experiment; 69th—at the end of the experiment). **(B)** VIP score bar plot of the detected VCs in piglet faecal sample groups (Green bars denote VCs, where VIP ≥ 1, while red bars denote VCs, where VIP < 1). **(C)** Volatile compound profiles of piglets’ faeces (volatile compound distribution in the control piglets’ group on day 69 vs. Pp group on day 69). **(D)** Volatile compound profiles of piglets’ faeces (volatile compound distribution in the control piglets’ group on day 69 vs. Pa group on day 69). **(E)** Volatile compound profiles of piglets’ faeces (volatile compound distribution in the Pa piglets’ group on day 69 vs. Pa group on day 69).

PLS-DA analysis (Figure 9A) clearly separated the C69d (C group on day 69) sample group from the other sample groups. Moreover, the Pp25 (day 25) and Pa25 (day 25) groups were clustered separately. The Pp and Pa group samples on day 69 are overlapping and exhibit much greater scattering. This higher scattering suggests higher variability in the individual VCs. In contrast, the C sample group showed the opposite trend, suggesting a much simpler VC profile in the faecal samples.

The VIP score analysis (Figure 9B) identified 19 analytes (VIP ≥ 1) as the most significant contributors to the variation between the sample groups. The top five VCs contributing most to group separation were pentadecanol, decyl methyl ketone, 3-phenylpropanol, hexadecanoic acid, and geranyl acetone. Other significant VCs included various acids (such as pentanoic, butanoic, 2-methylbutyric, octanoic, hydrocinnamic, nonanoic), aldehydes (such as cis-9-hexadecenal, (2E)-2-tetradecenal, dodecanal, octadecanal, (Z)-7-hexadecenal, non-(2E)-enal), phenolic compounds (p-cresol, 4-ethylphenol), alcohols (Z-2-dodecenol, cyclododecanol), esters (2-ethyl-3-hydroxyhexyl 2-methylpropanoate, hexadecanoic acid methyl ester), indoles (skatole), ketones (methyl octyl ketone), sesquiterpenes (*β*-gurjunene), alkanes, and their derivates (eicosane, hexadecene epoxide).

On day 69, the faecal VC profile of the C piglet group showed lower quantities of the following compounds, or they were not detected at all: cyclododecanol, Z-2-dodecenol, decyl methyl ketone, hexadecene epoxide, non-(2E)-enal, geranyl acetone, hexadecanoic acid, methyl octyl ketone, (2E)-2-tetradecenal, octadecanal, and 2-methylbutyric acid compared to the Pa group (Figure 9C). In contrast, the quantities of 11-dodecen-1-yl acetate, 1,3-di-tert-butylbenzene, dodecane, pentadecanal, pentadecanol, cis-9-hexadecenal, 3-phenylpropanol, and 4-ethylphenol were higher in the C group faeces compared to the Pa group.

When comparing the Pp and C groups’ faeces on day 69, the faecal VC profile of the C piglet group showed lower quantities of the following compounds, or they were not detected at all compared to the Pp group: geranyl acetone, hexadecanoic acid, decyl methyl ketone, 2,6-di-tert-butyl-4-methylphenol, (2E)-2-tetradecenal, Z-2-dodecenol, (Z)-7-hexadecenal, hexadecene epoxide, methyl octyl ketone, hexadecanoic acid methyl ester, octadecanal, cyclododecanol, eicosane, 2-methylbutyric acid (Figure 9D). In contrast, the quantities of 1,3-di-tert-butylbenzene, dodecane, 11-dodecen-1-yl acetate, pentadecanal, 2-ethyl-3-hydroxyhexyl 2-methylpropanoate, pentadecanol, cis-9-hexadecenal, dodecanal, 3-phenylpropanol, and hexanoic acid were higher in the C group’s faeces.

When comparing Pa and Pp groups’ faeces on day 69, at the end of the experiment, the faecal VC profile of the Pa piglet group showed lower quantities of the 2,6-Di-tert-butyl-4-methylphenol (Figure 9E).

## 4 Discussion

There is growing interest in using fermented feed for pigs. This study results showed that both treated groups (Pa and Pp) on day 69 showed higher body weights, in comparison with C group. However, significant differences in the FCR between the C, Pp, and Pa groups were not established. In the swine industry, the growth performance and health of weaned piglets are crucial for both animal productivity and economic profitability. Therefore, exploring healthier nutritional strategies to enhance the growth performance of weaned pigs is essential. Lactic acid bacteria with advantageous metabolic pathways for piglet fermented feed is essential for boosting nutrition, promoting gut health, increasing feed efficiency, and maintaining pigs’ general wellbeing. These bacteria may differ in the individual characteristics (tolerance to the acid environment, carbohydrate metabolism, etc.), the production of antimicrobial metabolites, vitamins and short-chain fatty acids as well as adaptation to the gut environment. Fermented feed is commonly inoculated with *Pediococcus* spp. and *L. plantarum* alone or in combination, because they produce significant amounts of lactic acid and reduce the feed’s pH (19). It was reported that *P. acidilactici* strain 72 N showed the greatest capacity to modify the quantity of lactobacilli and *Enterobacteriaceae* while also demonstrating the greatest ability to enhance intestinal structure in pigs (20). *P. acidilactici* synthesizes pediocin which is active against Gram-positive bacteria (21). It was found that *P. acidilactici* FT28 improved intestinal health and alleviated diarrheal symptoms in pigs by increasing the LAB population (22). By preserving the integrity of the gut epithelium, *P. pentosaceus* can reduce inflammation and regulate immunity (23). *Lactobacillus* is the main genera found in pigs’ digestive tracts and may improve gut metabolic capabilities (digestibility of nutrients and energy, energy content, and nitrogen retention), enhance weaning piglet immune systems, and preserve gut microbiota equilibrium (inhibit *Enterobacteriaceae* proliferation and *Escherichia coli*-induced intestinal permeability) (24). It was reported that in the digestive tracts of newborn piglets, *Lactobacillus reuteri* I5007 reduces harmful microorganisms and enhances the growth of a good bacteria (24). *Lactobacillus* strains MB 3123 (*L. johnsonii*), MB 3182 (*L. salivarius* group), and MB 3083 (*L. plantarum*) demonstrated high antimicrobial activity and organic acid production (19).

It has been reported that fermented feed helps alleviate stress in young pigs after weaning (25) and can improve the growth performance (26–28). It has also been reported that supplementing with fermented feed boosted the average daily gain of piglets between 28 and 42 days of age (29, 30). Including better digestibility (i.e., fermented) ingredients in piglet diets is crucial due to their immature digestive and immune systems (25). After fermentation, the macromolecular substances in feed are broken down into smaller molecules, making them easier for piglets to absorb and improving feed utilization (31). Fermenting feed, especially during the early growth stage of pigs, offers health benefits (32), suppress pathogen proliferation by lowering gastrointestinal pH (33), and improving gut microecology (26, 34). Additionally, solid fermented feed enhances palatability, which can increase feed intake in animals (35). Hu et al. (36) reported that a solid-state fermented diet increased the apparent total tract nutrient digestibility. The improved crude protein digestibility may result from the microbial degradation of antinutritional factors, such as cell wall components and phytates, as well as the breakdown of large protein molecules into smaller ones (36). Dietary supplementation with fermented feed has been shown to enhance nutrient digestion, promote feed intake, and ultimately improve the growth performance of weaned pigs (32). This complex effect of fermented feed material can explain the piglet weight results obtained in our experiment. However, the results may be related to the strain used. It has been reported that supplementation with 5% fermented soybean meal was found to improve FCR without affecting the blood profile (37). Supplementation of *Bacillus subtilis* to diets has been reported to significantly increase average daily gain (ADG) and reduce FCR in piglets, consistent with previous findings (38, 39). Another study showed that there was no significant difference in initial and final BWs between the *P. pentosaceus* CACC616 and control diet groups (23). However, none of the groups showed clinical symptoms, such as diarrhoea or mortality. The CACC616 group exhibited a lower FCR than the control group. The authors concluded that although *P. pentosaceus* CACC616 treatment led to increased average daily feed intake (ADFI) and improved FCR in weaned piglets after 26 days, the mechanisms through which supplementation with CACC616-based functional probiotics improves weaned piglet growth remain unclear. However, another studies have shown that diets supplemented with *P. pentosaceus* notably enhanced the growth performance of pigs while reducing the incidence of faecal noxious gases and diarrhoea (40, 41).

In our study analysis of blood parameters revealed no significant differences in IgA and IgM concentrations between the piglet groups. However, the highest IgG concentration was observed in the blood plasma of Pa piglets on day 69, although diet did not significantly affect IgG levels. Research by Grela et al. (42) suggests that fermented products enhance immunity in animals through both probiotic and prebiotic effects. It was found that piglets receiving *Bacillus subtilis* had significantly higher levels of IgA and IgG compared to those in the control and antibiotic-treated groups (43), and these outcomes align with earlier studies (44). It has been reported that *P. pentosaceus* can modulate immune function and reduce gut inflammation by maintaining the integrity of the gut epithelium (45, 46). Another blood plasma characteristics: ALT and AST are markers of hepatocellular damage. The liver, being the main organ for metabolism and detoxification, plays a crucial role in eliminating toxins, detoxifying blood, and removing unwanted drug metabolites that accumulate in other tissues (47, 48). Due to its distinct anatomical position, the liver is often exposed to circulating antigens, endotoxins, and microorganisms (49). In our study ALT levels were lower in both treated groups at the end of experiment, compared to the control group. However, diet was not found to significantly influence ALT or AST concentrations.

Also, no significant differences in faecal pH were observed between the groups when analyzing faecal indicators. However, on day 69, the Pp piglets had the highest faecal dry matter content, and both treated groups showed greater faecal texture hardness compared to the control group. Despite these findings, diet was not determined to be a significant factor for these parameters. It is also worth mentioning that no cases of diarrhea were reported in any of the groups by the end of the experiment. Microbiological analysis of piglet faecal samples revealed that all groups had higher TEC on day 69 compared to day 25. *Enterobacteriaceae* are part of the natural microflora in the guts of humans and animals. While they can lead to enteric diseases, many of these bacteria exist as commensals, with only a few species recognized as strict pathogens (50). On day 69, the highest LAB count was observed in the control and Pa groups compared to the Pp group. However, no significant differences in LAB counts were found between the Pp and Pa groups. Early intervention aimed at the gut microbiota could offer potential benefits in improving intestinal microbial balance (51–54). LAB are essential in preventing diseases by maintaining gut health (55, 56). *Lactobacillus*, specifically, are frequently used as probiotics after weaning because they are commensal bacteria that support gut immune function, help maintain the balance of gut microbiota and mitigate inflammation (57–60). In general, the microorganisms used in fermentation produce organic acids that significantly lower the feed’s pH (to 3.5–4.5), helping to inhibit the growth of pathogens in animals’ digestive tracts. Another benefit of using fermented feed is the presence of metabolites from microorganisms and LAB, and when these reach the animals’ intestines, they improve the intestinal microbiome, leading to more efficient energy utilization. However, despite that some LAB inhibits mold growth *in vitro* (61, 62), survival of individual species is thus often contingent on the niche created by metabolic activities of the others and yeast metabolites are benefit the LAB (63). By producing antimicrobial compounds (bacteriocins) and creating an acidic environment (lactic acid production), LAB prevents the growth or adhesion of pathogenic bacteria (64). By vying for binding sites on the intestinal epithelium or for nutrients in the intestine, LAB can suppress harmful bacteria (65). It was reported that *L. casei* reduced the quantity of *E. coli* that colonizes the gnotobiotic piglets’ jejunal mucosa (66). LAB have the ability to increase mucus secretion, which shields the intestinal lining and fosters a wholesome intestinal environment (64). By aiding in the breakdown of complex proteins and carbohydrates, LAB increases the availability of nutrients for absorption. Certain LAB strains exhibit immunomodulatory characteristics and the capacity to scavenge free radicals, which seems to be strain-dependent (64). In the small intestine of neonatal piglets, *Enterococcus faecium* EF1 produced a potent anti-inflammatory response (67).

Faecal microbiome composition showed that at the start point, i.e., the 25th day, the microbial composition in faeces was similar in all groups, with the dominance of *Ruminococcaceae*, *Christensenellaceae*, *Bacteroides*, and a moderate amount of *Lactobacillus.* Only Pp group has higher number of lactobacilli due to previous addition (0–25 days) of milk permeate to the feed of this group. The above-mentioned bacteria are known as normal predominant bacteria at this period of pigs’ life (1, 68). Lambo et al. (69) reported that using combinations and synergistic effect of different microorganisms (e.g., *Lactobacillus*, *Pediococcus*, *Streptococcus*, *Enterococcus*, *Lactococcus*, *Bifidobacteria*, etc.) can achieve greater positive effects on animal health and productivity than using a single microorganism. Wang et al. (70) observed that the synergistic effects of *Lactobacillus fermentum* and *Pediococcus acidilactici* improved intestinal health, more effectively reduced serum inflammatory factors, increased short-chain fatty acid production, and inhibited the proliferation of pathogenic microorganisms. Bacterial variety at this age showed a complex bacterial community without clearly dominant species but with clear increasing of alpha diversity during the growth of piglets. Such data is in coincidence with data obtained by other authors on the increasing bacterial variety in pigs’ gut from birth to the following growth stages (71, 72).

At the end of the experiment, on day 69, the predominant bacteria, including *Ruminococcaceae*, *Lachnospiraceae*, *Prevotellaceae*, *Lactobacillus*, and *Bacteroidales*, were observed in all animal groups; these species made about half amount from all faecal microbiomes. The above-mentioned bacteria are known as a core microbiota of pigs (73). The main difference between the groups at the end of experiment was observed in the prevalence of *Lactobacillus*, which surprisingly was significantly higher in the control group fed without fermented feed. Early-life seeding, stemming from maternal microbiota transmission and environmental exposure, establishes the foundational *Lactobacillus* populations that are crucial for neonatal gut health and disease resistance (1, 74, 75). Subsequently, in weaned piglets, nutrition, particularly the composition of dietary carbohydrates, and fermented feed material, become primary drivers of *Lactobacillus* abundance and diversity. The reason of such increase of *Lactobacillus* in this animal group remain unknown. The data on the dynamics of *Lactobacillus* after the weaning period in different studies are contradictory. Some research indicates that *Lactobacillus* populations remain consistent with pre-weaning levels (76, 77), while other authors report a significant increase in faecal samples during the post-weaning period (78). In our study, the variety of *Lactobacillus* species detected in faecal content of pigs included *Limosilactobacillus mucosae*, *Lactobacillus johnsonii*, *Limosilactobacillus reuteri*, *Limosilactobacillus vaginalis*, *Lactobacillus delbrueckii and Lactobacillus gallinarum.* However, most of the reads remained unidentified up to the species level. Despite the higher numbers of *Lactobacillus* in the control group, the alpha diversity in this group was lowest in comparison with groups fed with fermented feed. It is known that healthy microbial community often demonstrates high taxonomic diversity, high microbial gene richness, and stable core microbiota (79). As body weight was higher in the pigs fed with fermented feed, it may be assumed that a single bacterial taxon, even if it is known as beneficial, is unable to provide better clinical status, higher immunoglobulin level or better zootechnical parameters. It also should be noted that the faecal microbiome does not provide clear insight into microbial composition in separate parts of the gastrointestinal tract, therefore microbial indices in faeces should not be treated as the key parameter within this study.

**Figure 10:**
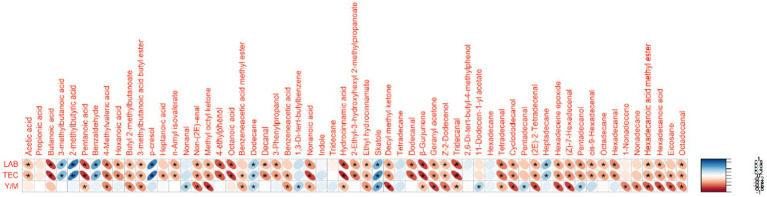
Correlations between faeces microbiological parameters and volatile compounds (LAB, lactic acid bacteria; TEC, total enterobacteria count; Y/M, yeast and mold). *Denotes statistically significant correlations (*p* ≤ 0.05).

It has been reported that faecal VCs may serve as biomarkers of gastrointestinal functionality, as changes in microbial and cellular metabolism can lead to variations in the VC profile of faeces (80). The faecal samples from the Pp and Pa groups on day 69 showed significantly greater variability in individual VCs compared to the control group. It could be seen from the Figure 10 that there are significant correlations between separate VC and faeces microbiological parameters. Out of the 59 VCs identified in piglet faeces, significant correlations were found between 43 VCs and LAB count, between 47 VCs and TEC, and between 34 VCs and yeast and mold count. In our previous studies, similar tendencies were established (7). On day 69, butyl 2-methylbutanoate, 3-methylbutanoic acid butyl ester, non-(2E)-enal, methyl octyl ketone, benzeneacetic acid methyl ester, ethyl hydrocinnamate, decyl methyl ketone, geranyl acetone, Z-2-dodecenol, cyclododecanol, (2E)-2-tetradecenal, hexadecene epoxide, (Z)-7-hexadecenal, nonadecane, hexadecanoic acid methyl ester, hexadecanoic acid, eicosane, and octadecanal were only detected in treated groups (Pa and Pp) faeces. Additionally, both treated groups showed higher body weights at the end of the experiment. Butyl 2-methylbutanoate is a fatty acid ester, which is a carboxylic ester derivative of a fatty acid. The synthesis of this ester may be mediated by a bacterial esterase, such as the one present in *E. coli* (81) or by enzyme(s) derived from the intestinal mucosa (82). There is a paucity of literature in this area, but reports suggest that the intestinal mucosa may be a major site for the synthesis of fatty acid ethyl esters (83). Although esters naturally occur in many feedstuffs, they are expected to be primarily hydrolyzed by the piglet’s digestive system. Geranyl acetone is a monoterpene ketone, where an (E)-geranyl group is bonded to one of the alpha-methyl groups of acetones. It is a component of essential oils from various plants. Methylketones can be produced by many bacterial species and by fungi, which convert the respective alkanoic acids, however, some compounds in this group may also have dietary origins (84). Aliphatic aldehydes can result from bacterial activity or autooxidation of unsaturated fatty acids, so their upregulation may be associated with a high-fat diet (85). Alkanes of various chain lengths can originate from plants and be excreted in manure or faeces due to limited absorption in the intestine. Alternatively, like aldehydes, they can also be produced through lipid peroxidation (86, 87).

Finally, greater variability in individual VCs compared to the control group could be associated with the greater variability of bacterial species in the treated group’s faeces, as well as with deeper nutrient degradation.

## 5 Conclusion

This study showed that continuing to feed piglets (from suckling to weaning and into the later stages) with FFM may be beneficial for enhancing their growth performance and health parameters. Both treated groups (Pa and Pp) on day 69 showed higher body weights compared to the control group. Additionally, the control and Pa groups on day 69 had higher blood plasma IgM concentrations and lower ALT levels. Both treated groups exhibited higher faecal texture hardness compared to the control group. The faeces from the treated groups showed greater bacterial diversity. Furthermore, samples from the Pp and Pa groups on day 69 showed significantly greater variability in individual faecal VCs compared to the control group, with significant correlations between individual VCs and faecal microbiological parameters. Finally, the greater bacterial diversity and variability in individual VCs in the treated groups could be linked to enhanced nutrient degradation and absorption as well as higher body weights of the piglets.

## Data Availability

The original contributions presented in the study are included in the article/Supplementary material, further inquiries can be directed to the corresponding author/s. Sequences were deposited in the NCBI database under accession number PRJNA1205751.

## Supplementary material

SUPPLEMENTARY TABLE S1 Carbohydrate metabolism, gas production, tolerance to temperature (10, 30, 37, and 45°C) and low pH conditions (pH 2.5 for 2 h) of the *Lb. plantarum* LUHS122, *Lb. casei* LUHS210, *Lb. curvatus* LUHS51, and *Lb. paracasei* LUHS244 strains.

SUPPLEMENTARY TABLE S2 Inhibition zones of the tested lactic acid bacteria (LAB) strains against pathogenic opportunistic microorganisms.

SUPPLEMENTARY TABLE S3 Antimicrobial activities of the tested lactic acid bacteria (LAB) strains against pathogenic opportunistic microorganisms in liquid medium (+ indicates pathogen growth; − indicates that pathogen growth was not observed).

SUPPLEMENTARY TABLE S2.1 Volatile compound profiles of piglets’ faeces.
