# Supplementary material for: Modeling the weaning diet of piglets with fermented feed material: effects on growth performance and health parameters
Source: Front Vet Sci. 2025 Jul 3;12:1616209. doi: 10.3389/fvets.2025.1616209 (PMC12267039; doi:10.3389/fvets.2025.1616209)
Supplement: SUPPLEMENTARY TABLE S1 — Carbohydrate metabolism, gas production, tolerance to temperature (10, 30, 37, and 45°C) and low pH conditions (pH 2.5 for 2 h) of the Lb. plantarum LUHS122, Lb. casei LUHS210, Lb. curvatus LUHS51, and Lb. paracasei LUHS244 strains. [file Data_Sheet_1.pdf]

Supplementary Material

Supplementary Tables

Table S1. Carbohydrate metabolism, gas production, tolerance to temperature (10, 30, 37 and 45 °C) and low pH conditions (pH 2.5 for 2 h) of the *Lb. plantarum* LUHS122, *Lb. casei* LUHS210, *Lb. curvatus* LUHS51, and *Lb. paracasei* LUHS244 strains.

**Parameters**	*L. plantarum* No. 122	*L. curvatus* No. 51	*L. casei* No. 210	*L. paracasei* No. 244
Glicerol	–	–	–	–
D-arabinose	–	–	–	–
L-arabinose	+++	+++	–	–
D-ribose	+++	+++	+++	+++
D-xylose	–	–	–	–
L-xylose	–	–	–	+++
D-adonitol	–	–	–	+
Methyl-ßd-xYlopiranoside	–	–	–	–
D-galactose	+++	+++	+++	+++
D-glucose	+++	+++	+++	+++
D-fructose	+++	+++	+++	+++
D-mannose	+++	+++	+++	+++
L-sorbose	–	–	–	–
L-rhamnose	+	–	–	+++
Dulcitol	–	–	+++	+++
Inositol	–	–	–	–
D-mannitol	+++	+++	+++	+++
D-sorbitol	+++	+++	+++	+++
Methyl-αD-mannopyranoside	+++	+	–	–
Methyl-αD-glucopyranoside	–	–	+++	+++
N-acetylglucosamine	+++	+++	+++	+++
Amigdalin	+++	+++	+++	+++
Arbutin	+++	+++	+++	+++
Esculin	+++	+++	+++	+++
Salicin	+++	+++	+++	+++
D-cellobiose	+++	+++	+++	+++
D-maltose	+++	+++	++	+++
D-lactose	+++	+++	–	+++
D-melibiose	+++	–	–	–
D-saccharose	+++	+++	+++	+++
D-trehalose	+++	+++	+++	+++
Inulin	–	–	++	+++

D-melezitose	+++	+++	+++	+++
D-raffinose	+++	−	−	−
Amidon	−	−	−	−
Glycogen	−	−	−	−
Xylitol	−	−	−	−
Gentiobiose	++	++	++	+++
D-turanose	+++	+++	+++	+++
D-tagatose	+++	−	+++	+++
D-fucose	−	−	−	−
L-fucose	−	−	−	−
D-arabitol	−	−	−	−
L-arabitol	−	−	−	−
Potassium gluconate	+	+	+	++
Potassium 2-ketogluconate	−	−	−	−
Potassium 5-ketogluconate	−	−	−	−
Gas production (+/−)	−	−	−	−
Temperature tolerance	−	−	+	−
	++	+	+++	++
	+	+	+++	++
	+	−	+	−
pH 2.5	8.43 ± 0.3	8.31 ± 0.2	8.47 ± 0.3	9.41 ± 0.2
	5.72 ± 0.2	3.5 ± 0.1	8.36 ± 0.2	9.29 ± 0.1

Interpretation of lactic acid bacteria (LAB) growth in API 50 CH system and API 20 E system: +++ = strong growth (yellow); ++ = moderate growth (green); + = weak growth (dark green); − = no growth (blue); n.d. = not determined.

Table S2. Inhibition zones of the tested lactic acid bacteria (LAB) strains against pathogenic opportunistic microorganisms.

LAB strains	Inhibition zones, mm														
	Pathogenic and opportunistic bacteria strains														
	Klebsiella pneumoniae	*Salmonella enterica* 24 SPn06	*Pseudomonas aeruginosa* 17-331	*Acinetobacter baumannii* 17-380	*Proteus mirabilis*	*MRSA* M87fox	*Enterococcus faecalis* 86	*Enterococcus faecium* 103	*Bacillus cereus* 18 01	*Streptococcus mutans*	*Enterobacter cloacae*	*Citrobacter freundii*	*Streptococcus epidermis*	*Staphylo-coccus haemolyticus*	*Pasteurella multocida*
LUHS 122	12.1 ±0.3	13.2 ±0.3	17.2 ±0.3	13.1 ±0.2	14.6 ±0.2	14.0 ±0.2	14.0 ±0.4	15.4 ±0.2	16.0 ±0.2	21.0 ±0.2	13.2 ±0.3	16.0 ±0.2	20.0 ±0.2	18.1 ±0.2	25.1 ±0.2
LUHS 210	13.2 ±0.1	13.4 ±0.3	12.3 ±0.4	14.5 ±0.3	16.2 ±0.2	15.3 ±0.4	15.2 ±0.2	12.2 ±0.3	15.0 ±0.2	19.5 ±0.3	15.1 ±0.2	16.3 ±0.2	18.4 ±0.3	19.0 ±0.4	25.3 ±0.4
LUHS 51	12.1 ±0.3	12.0 ±0.2	15.5 ±0.3	14.3 ±0.1	11.3 ±0.1	12.0 ±0.2	12.1 ±0.3	12.1 ±0.2	9.0 ±0.2	18.1 ±0.1	12.1 ±0.3	12.0 ±0.4	14.0 ±0.2	14.1 ±0.3	20.4 ±0.3
LUHS 244	10.2 ±0.2	11.1 ±0.4	14.5 ±0.2	15.0 ±0.2	13.6 ±0.2	14.1 ±0.4	12.1 ±0.2	13.2 ±0.4	20.1 ±0.	24.4 ±0.3	14.0 ±0.2	15.0 ±0.2	17.0 ±0.2	16.0 ±0.4	43.5 ±0.5

Data expressed as mean values (n = 3) ± SD; SD – standard deviation.

MRSA – Methicillin-resistant *Staphylococcus aureus*; LAB – lactic acid bacteria; *Lb. plantarum* LUHS122; *Lb. casei* LUHS210; *Lb. curvatus* LUHS51; *Lb. paracasei* LUHS244.

Table S3. Antimicrobial activities of the tested lactic acid bacteria (LAB) strains against pathogenic opportunistic microorganisms in liquid medium (+ indicates pathogen growth; - indicates that pathogen growth was not observed).

LAB strains	*Klebsiella pneumoniae*	*Salmonella enterica* 24 SPn06	*Pseudomonas aeruginosa* 17-331	*Acinetobacter baumannii* 17-380	*Proteus mirabilis*	*MRSA* M87fox	*Enterococcus faecalis* 86	*Enterococcus faecium* 103	*Bacillus cereus* 18 01	*Streptococcus mutans*	*Enterobacter cloacae*	*Citrobacter freundii*	*Streptococcus epidermis*	*Staphylococcus haemolyticus*	*Pasteurella multocida*	Number of the inhibited patho-gens
Experimental design: 0.5 mL LAB + 0.1 mL pathogen																
LUHS122	-	-	-	-	-	-	-	-	-	-	+	-	-	-	-	14
LUHS210	-	-	-	-	-	-	-	-	-	-	-	-	-	-	-	15
LUHS51	+	-	-	-	-	+	-	-	-	-	-	-	-	-	-	13
LUHS244	-	+	-	+	-	+	-	-	-	-	-	-	-	-	-	12
Pathogen control	+	+	+	+	+	+	+	+	+	+	+	+	+	+	+	-
LAB control	+	+	+	+	+	+	+	+	+	+	+	+	+	+	+	-
Experimental design: 1.0 mL LAB + 0.1 mL pathogen																
LUHS122	-	-	-	-	-	-	-	-	-	-	-	-	-	-	-	15
LUHS210	-	-	-	-	-	-	-	-	-	-	-	-	-	-	-	15
LUHS51	-	-	-	-	-	+	-	-	-	-	-	-	-	-	-	14
LUHS244	-	+	-	-	-	-	-	-	-	-	-	-	-	-	-	14
Pathogen control	+	+	+	+	+	+	+	+	+	+	+	+	+	+	+	-
LAB control	+	+	+	+	+	+	+	+	+	+	+	+	+	+	+	-

MRSA – Methicillin-resistant *Staphylococcus aureus*; *MRSA* – Methicillin-resistant *Staphylococcus aureus*; LAB – lactic acid bacteria; *Lb. plantarum* LUHS122; *Lb. casei* LUHS210; *Lb. curvatus* LUHS51; *Lb. paracasei* LUHS244.
