# Supplementary material for: Modeling the weaning diet of piglets with fermented feed material: effects on growth performance and health parameters
Source: Front Vet Sci. 2025 Jul 3;12:1616209. doi: 10.3389/fvets.2025.1616209 (PMC12267039; doi:10.3389/fvets.2025.1616209)
Supplement: SUPPLEMENTARY TABLE S2.1 — Volatile compound profiles of piglets’ faeces. [file Data_Sheet_2.pdf]

Supplementary Material

Supplementary Tables

Table S2.1. Volatile compound profiles of piglets' faeces.

Volatile compound	C25d	Pa25d	Pp25d	C69d	Pa69d	Pp69d
Acetic acid	1.82 ± 1.100	2.06 ± 1.04	1.92 ± 1.22	4.46 ± 1.14	5.07 ± 1.73	4.50 ± 0.972
Propionic acid	2.91± 1.56	4.88 ± 1.63	2.35 ± 0.744	6.65 ± 4.10	6.88 ± 3.54	6.31 ± 2.38
Butanoic acid	2.63 ± 4.16	7.24 ± 6.96	2.47 ± 3.45	56.5 ± 18.4	49.0 ± 28.0	50.2 ± 21.7
3-methylbutanoic acid	4.46 ± 2.77	5.56 ± 2.61	4.36 ± 1.56	4.90 ± 1.80	6.09 ± 3.00	7.40 ± 4.94
2-methylbutyric acid	37.9 ± 16.4	39.6 ± 15.4	40.4 ± 14.8	20.6 ± 12.6	42.3 ± 16.3	51.5 ± 32.4
Pentanoic acid	6.4 ± 8.4	8.6 ± 10.7	11.9 ± 9.52	61.9 ± 17.9	58.8 ± 19.8	56.8 ± 11.7
Benzaldehyde	1.57 ± 0.492	0.923 ± 0.805	2.01 ± 0.498	0.426 ± 0.148	0.584 ± 0.26	1.02 ± 0.754
4-Methylvaleric acid	ND	0.212 ± 0.508	ND	0.669 ± 0.283	1.09 ± 0.766	1.39 ± 0.536
Hexanoic acid	3.28 ± 3.65	1.23 ± 0.906	4.11 ± 5.92	21.2 ± 23.6	11.0 ± 9.98	4.92 ± 5.81
Butyl 2-methylbutanoate	ND	ND	ND	ND	0.076 ± 0.094	0.047 ± 0.082
3-methylbutanoic acid butyl ester	ND	ND	ND	ND	0.097 ± 0.133	0.082 ± 0.110
p-cresol	56.1 ± 21.6	63.3 ± 17.6	52.7 ± 8.94	38.7 ± 17.3	65.1 ± 14.8	66.1 ± 29.3
Heptanoic acid	ND	ND	0.541 ± 1.22	3.50 ± 5.90	2.06 ± 2.60	0.292 ± 0.714
n-Amyl isovalerate	ND	ND	ND	0.585 ± 0.900	0.249 ± 0.262	0.202 ± 0.268
Nonanal	0.263 ± 0.117	0.239 ± 0.098	0.196 ± 0.049	0.268 ± 0.076	0.192 ± 0.028	0.209 ± 0.042
Non-(2E)-enal	ND	ND	ND	ND	0.111 ± 0.036	0.219 ± 0.141
Methyl octyl ketone	ND	ND	ND	ND	0.127 ± 0.072	0.115 ± 0.060
4-ethylphenol	0.009 ± 0.022	ND	0.014 ± 0.043	3.31 ± 2.30	1.10 ± 0.759	1.82 ± 2.05
Octanoic acid	0.019 ± 0.047	ND	0.028 ± 0.059	0.302 ± 0.149	0.180 ± 0.081	0.166 ± 0.076
Benzeneacetic acid methyl ester	ND	ND	ND	ND	0.046 ± 0.071	0.011 ± 0.017
Dodecane	0.527 ± 0.164	0.305 ± 0.288	0.488 ± 0.186	0.198 ± 0.064	0.083 ± 0.020	0.081 ± 0.018
Decanal	0.059 ± 0.022	0.031 ± 0.017	0.060 ± 0.021	0.160 ± 0.053	0.135 ± 0.046	0.102 ± 0.023
3-Phenylpropanol	ND	ND	ND	0.135 ± 0.124	ND	ND
Benzeneacetic acid	0.486 ± 0.439	0.470 ± 0.554	0.641 ± 0.531	1.14 ± 0.795	2.18 ± 0.629	2.76 ± 1.84
1,3-Di-tert-butylbenzene	0.444 ± 0.193	0.194 ± 0.270	0.408 ± 0.190	0.254 ± 0.104	ND	ND
Nonanoic acid	0.026 ± 0.038	0.010 ± 0.026	0.066 ± 0.064	0.198 ± 0.073	0.219 ± 0.110	0.202 ± 0.059
Indole	1.25 ± 1.56	2.29 ± 2.37	0.450 ± 0.246	1.19 ± 0.963	1.91 ± 1.38	1.91 ± 0.943
Tridecane	0.126 ± 0.134	0.048 ± 0.086	0.119 ± 0.085	0.136 ± 0.091	0.182 ± 0.075	0.195 ± 0.035
Hydrocinnamic acid	0.068 ± 0.112	0.013 ± 0.030	0.095 ± 0.147	1.81 ± 0.880	1.76 ± 0.618	2.00 ± 0.658
2-Ethyl-3-hydroxyhexyl 2-methylpropanoate	0.020 ± 0.022	0.005 ± 0.008	0.032 ± 0.015	0.102 ± 0.035	0.053 ± 0.023	0.039 ± 0.021

Ethyl hydrocinnamate	ND	ND	ND	ND	0.086 ± 0.150	0.058 ± 0.142
Skatole	25.6 ± 12.4	21.4 ± 7.57	19.7 ± 8.90	14.6 ± 5.18	11.0 ± 4.79	12.6 ± 5.76
Decyl methyl ketone	ND	ND	ND	ND	0.176 ± 0.057	0.197 ± 0.029
Tetradecane	0.134 ± 0.046	0.068 ± 0.042	0.161 ± 0.040	0.144 ± 0.046	0.124 ± 0.017	0.132 ± 0.033
Dodecanal	0.147 ± 0.097	0.022 ± 0.034	0.197 ± 0.153	0.850 ± 0.348	0.465 ± 0.179	0.389 ± 0.107
β-Gurjunene	0.001 ± 0.005	ND	ND	0.085 ± 0.041	0.106 ± 0.045	0.133 ± 0.033
Geranyl acetone	ND	ND	ND	ND	0.054 ± 0.025	0.068 ± 0.014
Z-2-Dodecenol	ND	ND	ND	ND	0.566 ± 0.224	0.490 ± 0.222
Tridecanal	0.087 ± 0.060	0.024 ± 0.027	0.088 ± 0.048	0.453 ± 0.160	0.637 ± 0.343	0.544 ± 0.207
2,6-Di-tert-butyl-4-methylphenol	0.454 ± 0.366	0.034 ± 0.044	0.587 ± 0.324	0.129 ± 0.078	0.217 ± 0.069	0.879 ± 0.204
11-Dodecen-1-yl acetate	0.641 ± 0.734	0.061 ± 0.036	0.516 ± 0.387	1.27 ± 0.537	ND	ND
Hexadecane	0.246 ± 0.121	0.108 ± 0.044	0.296 ± 0.105	0.207 ± 0.100	0.319 ± 0.121	0.246 ± 0.132
Tetradecanal	0.439 ± 0.254	0.157 ± 0.057	0.434 ± 0.240	1.36 ± 0.557	2.97 ± 1.97	2.13 ± 0.772
Cyclododecanol	ND	ND	ND	ND	1.32 ± 0.43	1.10 ± 0.391
Pentadecanal	0.664 ± 0.559	0.159 ± 0.080	0.568 ± 0.282	1.54 ± 0.683	0.492 ± 0.129	0.434 ± 0.153
(2E)-2-Tetradecenal	ND	ND	ND	ND	2.07 ± 1.09	1.55 ± 0.364
Heptadecane	0.379 ± 0.152	0.219 ± 0.053	0.534 ± 0.224	0.246 ± 0.092	0.359 ± 0.066	0.270 ± 0.092
Hexadecene epoxide	ND	ND	ND	ND	1.07 ± 0.425	0.956 ± 0.224
(Z)-7-Hexadecenal	ND	ND	ND	ND	0.248 ± 0.215	0.281 ± 0.106
Pentadecanol	0.106 ± 0.051	0.072 ± 0.043	0.092 ± 0.047	0.715 ± 0.495	ND	ND
cis-9-Hexadecenal	0.063 ± 0.045	0.065 ± 0.059	0.065 ± 0.039	0.450 ± 0.354	ND	ND
Octadecane	0.066 ± 0.024	0.041 ± 0.024	0.102 ± 0.055	0.305 ± 0.343	0.217 ± 0.133	0.166 ± 0.052
Hexadecanal	0.664 ± 0.322	0.370 ± 0.142	0.525 ± 0.235	1.68 ± 1.02	1.27 ± 0.363	1.30 ± 0.486
1-Nonadecene	ND	ND	ND	ND	ND	0.004 ± 0.010
Nonadecane	ND	ND	ND	ND	0.254 ± 0.434	0.153 ± 0.203
Hexadecanoic acid methyl ester	ND	ND	ND	ND	0.091 ± 0.086	0.062 ± 0.046
Hexadecanoic acid	ND	ND	ND	ND	0.485 ± 0.253	0.431 ± 0.102
Eicosane	ND	ND	ND	ND	0.672 ± 0.783	0.553 ± 0.443
Octadecanal	ND	ND	ND	ND	0.106 ± 0.069	0.111 ± 0.038

C – control group – fed with basal non-fermented diet, previously received full-fledged combined pre starter feed for piglets PANTO®; Pp and Pa groups – previously, additionally to traditional diet from the 7th day of life received fermented with Pp and Pa milk permeate (groups Pp and Pa, respectively) and during the continuous experiment additionally received fermented with *Lb. plantarum, Lb. casei, Lb. curvatus,* and *Lb. paracasei* feed material; 25th – at the beginning of the experiment; 69th – at the end of the experiment.
